# Discovery of microbial glycoside hydrolases *via* enrichment and metaproteomics

**DOI:** 10.1039/d5cb00049a

**Published:** 2025-09-25

**Authors:** Jitske M. van Ede, Suzanne van der Steen, Geert M. van der Kraan, Mark C. M. van Loosdrecht, Martin Pabst

**Affiliations:** a Department of Biotechnology, Delft University of Technology Delft The Netherlands m.pabst@tudelft.nl; b Genencor International B.V., International Flavors & Fragrances Oegstgeest The Netherlands

## Abstract

The immense microbial diversity on Earth represents a vast genomic resource, yet discovering novel enzymes from complex environments remains challenging. Here, we combine a microbial enrichment with metagenomics and metaproteomics to facilitate the identification of microbial glycoside hydrolases that operate under defined conditions. We enriched microbial communities on the carbohydrate polymer pullulan at elevated temperatures under acidic conditions. Pullulan is a natural polysaccharide composed of maltotriose units linked by α-1,6-glycosidic bonds. Pullulan, along with its hydrolyzing enzymes, has broad applications across various industries. The enrichment inocula were sampled from thermophilic compost and from soil from the bank of a pond. In both cases, *Alicyclobacillus* was identified as the dominant microorganism. Metaproteomic analysis of the enriched biomass and secretome enabled the identification of several pullulan-degrading enzyme candidates from this organism. These enzymes were absent in the metagenomic analysis of the initial inoculum, which is highly complex with a wide diversity of species. This underscores the effectiveness of combining microbial enrichment with multi-omics for uncovering novel enzymes and sequence variants that operate under defined conditions from complex microbial environments.

## Abstract 2

Targeted discovery of glycoside hydrolases from environmental sources through enrichment, metagenomics, and cellular location-informed metaproteomics.

## Graphical abstract

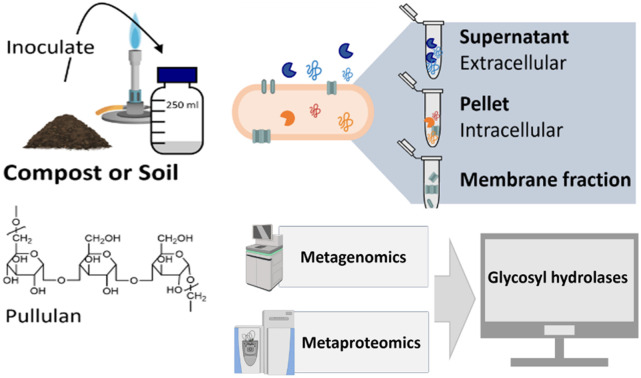

## Introduction

Enzymes are natural biocatalysts with great potential to advance sustainable processes in biotechnology and industry. Not only are they biodegradable, but they also have the potential to conduct conversions under ambient conditions, and reduce the use of fossil fuels and chemical waste. Moreover, enzymes provide complex molecules that are challenging to produce *via* chemical synthesis alone.^1,2^ Enzymes are already extensively employed in industry, with hydrolases being the most prevalent class of enzymes in use.^3^ Hydrolases can modify biopolymers, altering their properties, or break them down into smaller fragments. A vast array of applications exists for (modified) biopolymers. For example, carbohydrate biopolymers are widely applied in the food industry, nutraceuticals, and care products. Cellulose, obtained from plant cell walls, is used as a stabilizer and thickener in various food formulations.^4^ Chitosan, derived from crustacean shells, exhibits antimicrobial properties, making it a valuable component in dietary supplements.^5^ Another example is pullulan, a natural polysaccharide produced by the fungus *Aureobasidium pullulans*, which is highly valued in the food and pharmaceutical industries for its ability to form edible, non-toxic films and coatings.^6,7^ Its applications include encapsulating food flavours and fragrances, improving product shelf life, and acting as a binder in orally dissolvable films for pharmaceuticals, demonstrating its versatility and biocompatibility.^8^ Furthermore, also the hydrolysing enzymes are used in industrial applications. For example, pullulanases are essential in the starch-processing industry, where they are used to hydrolyze pullulan into maltotriose units, thereby enhancing the efficiency of starch liquefaction and saccharification processes. Their use significantly improves the production of glucose and maltose syrups, providing a higher yield and specificity compared to conventional methods.^9^ Hydrolases are also employed in cleaning processes, such as laundry. These enzymes facilitate the breakdown of polymers, thereby boosting cleaning efficiency. Additionally, their application offers environmental benefits by enabling lower washing temperatures, shorter wash times, and reduced water usage.^10,11^

Pullulan represents a polysaccharide that consists of maltotriose units, in which three glucoses are linked *via* an α-1,4-linkage, and these maltotriose units are linked *via* α-1,6-glycosidic bonds.^12^ Pullulan degrading enzymes can be divided into two main groups: pullulanases (type I and type II) and pullulan hydrolases (types I, II and III). Pullulanases cleave the α-1,6 bond, thereby converting pullulan into maltotriose. In addition, pullulanases are also active against other sugar polymers, including starch and glycogen. While pullulanase type I can only cleave the α-1,6-linkages of these sugar polymers, pullulanase type II can cleave both the α-1,6- and α-1,4-linkages. Pullulan hydrolases are further divided into three types. Type I (neopullulanase) and type II (isopullulanase) cleave the α-1,4-linkage of pullulan, thereby forming panose and isopanose, respectively. Type III has the ability to cleave both the α-1,4- and α-1,6-linkages of pullulan, forming panose, maltotriose, maltose and glucose.^13^ In general, the optimal pH of pullulan-degrading enzymes varies widely, ranging from pH 3 to 11, although the majority exhibit optimal activity between pH 5 and 8. Similarly, their optimal temperatures span a broad range from 30 °C to 105 °C (SI, Fig. S5).^13^ Certain enzyme groups, such as pullulanase types I and II, include members active across this entire temperature and pH spectrum. The so far identified pullulan hydrolases of types I and II seem to occupy a more narrow space, with pH optima in the range of 5–8 for type I and 3.5–5 for type II. Type III pullulan hydrolases have only been identified in two archaeal species, both exhibiting high optimal temperatures of 95 °C and 97.5 °C, and optimal pH values of 6.5 and 3.5, respectively (SI, Fig. S5).^13^

Although a wide variety of enzymes are utilized in various industries, the quest for new enzymes – including pullulanases – with improved features is ongoing.^13^ There is currently a focused effort on employing advanced protein/enzyme optimization techniques, such as artificial intelligence, directed evolution, and intelligent design, to improve enzymes.^14–20^ However, despite progress in protein engineering and other modification strategies, these approaches do not always yield the anticipated results due to the complex relationship between the structure and function of proteins.^21^ Fortunately, a remarkable diversity of microorganisms inhabit our planet, providing us with a vast genetic and therefore biocatalytic resource.^2^ Exploring this natural diversity with sophisticated enzyme discovery methods presents a promising alternative for finding enzymes with the desired traits or for identifying templates for further engineering.

Conventional methods for mining genetic resources rely on cultivating microorganisms in the laboratory, after which DNA is extracted and sequenced if culturing is successful.^22,23^ After the genome is assembled, it can be mined for genetic targets of interest. Nevertheless, this approach has limitations, namely, most microorganisms cannot be grown as a pure culture under conditions that prevail in laboratory environments. This limitation is better known as “the great plate anomaly”. It is expected that only about 1% of the microorganisms in a sampled environment can be found in a lab after enrichment.^24–26^ A way to avoid this problem and achieve culture independence is by using metagenomics.^27^ Over the past years, whole metagenome sequencing has become a popular method to sequence the entire metagenome from natural microbial communities.^28^ Currently, there are two main metagenomic strategies: the sequence-based and the function-based approach.^29^ The function-based approach involves constructing a genomic library from environmental DNA, followed by screening individual genes in a cultivable host cell for desired activities.^28–30^ While this method enables the discovery of entirely novel enzymes, it is limited by high costs and the requirement of high-throughput activity assays.^2^ In contrast, the sequence-based approach depends on sequence similarity to known templates,^29,30^ which restricts its ability to identify enzymes with novel functions.^2^

More recent strategies include microbial enrichment cultures followed by multi-omics analysis of the cultures (genomics and proteomics, supported by advanced bioinformatics tools).^31^ These strategies first enrich for certain organisms that possess an enzyme with a desired function. This route is only feasible when the enzyme plays a crucial role in the growth of the organism, *e.g.* is involved in the carbon or nitrogen source utilization.^32^ For instance, in pursuit of hydrolytic enzymes that cleave specific polymers, an enrichment with the polymer as the sole carbon source can be performed. This ensures that only microorganisms that can hydrolyse and utilize the polymer hydrolysis products will grow.^33^ Moreover, the conditions for enriching microbes can be tuned towards the conditions in which the enzymes should be active (*e.g.* temperature, pH and salinity). Subsequently, instead of only employing metagenomics to mine for the enzymes of interest, a more streamlined approach is to also integrate proteomics into the workflow.^31,34^ Advantageously, metaproteomics allows identification of the actually expressed enzymes, as well as their cellular location, such as cytosolic, membrane-bound, or secreted.^35,36^ This greatly simplifies genomic data and it supports identification of novel enzyme candidates or those with enhanced properties. It also provides template sequences for further engineering to optimize desired properties.^37^ In summary, this study demonstrates the discovery of novel pullulan-degrading enzymes that operate under defined conditions, by combining microbial enrichment with metagenomics and metaproteomics.

## Methods

### Microbial enrichment

Organic matter from compost was obtained from a compost pile in Waddinxveen (further referred to as “compost”), The Netherlands. Soil was collected from two locations in Delft, The Netherlands (at approximately 51°59′36.3′′N 4°22′23.6′′E and 51°59′37.5′′N 4°22′34.0′′E) and combined (further referred to as “soil”). The enrichments were performed under sterile conditions (to ensure only microbial diversity from the sampled sources is enriched) in 250 mL shake flasks with screw caps. The growth medium contained 0.5 g L^−1^ KH_2_PO_4_, 3 g L^−1^ (NH_4_)_2_SO_4_, 0.2 g L^−1^ MgSO_4_·7H_2_O, 10 ml L^−1^ MD-VS ATCC vitamin supplement and 2.5 ml L^−1^ trace element solution (15 g L^−1^ EDTA (Titriplex III), 4.5 g L^−1^ ZnSO_4_·7H_2_O, 0.84 g L^−1^ MnCl_2_·2H_2_O, 0.30 CoCl_2_·6H_2_O, 0.20 g L^−1^ CuSO_4_·5H_2_O, 0.40 g L^−1^ Na_2_MoO_4_·2H_2_O, 4.5 g L^−1^ CaCl_2_·2H_2_O, 3.0 g L^−1^ FeSO_4_·7H_2_O, 1.0 g L^−1^ H_3_BO_3_ and 0.10 g L^−1^ KI). Pullulan was added to a final concentration of 10 g L^−1^ and the pH of the medium was adjusted to 4.5 with 2 M HCl. Finally, the medium was sterilized in an autoclave at 110 °C for 30 min and subsequently stored at 4 °C until use. 50 mL of the growth medium was transferred to a 250 mL sterile flask. The medium was inoculated by either 0.5 g of compost, further referred to as “compost enrichment”, or by 0.5 g of soil, further referred to as “soil enrichment”. In addition, a control culture without inoculum was included. The enrichments were kept at 57 °C, 100 rpm in the dark in an incubator hood (TH 15, Edmund Buhler GmbH). The pH was monitored twice a day using a pH probe (826 pH mobile, Metrohm) and if necessary adjusted back to pH 4.5 using 0.2 M KOH or 0.2 M HCl.

No organic buffer was used to prevent it from becoming a potential substrate for microbial growth. All steps were performed next to a Bunsen burner to maintain sterile conditions. The enrichments were transferred twice in a 1 : 1000 (v/v) ratio to fresh medium when almost all pullulan was consumed, after 3 and 4 days, respectively. After the second transfer, the OD_600_ (using an Ultrospec 10 cell density meter from Biochrom) and the pullulan consumption (measured by HPLC analysis) was monitored daily, while the pH was still measured twice a day. The microbial enrichments were analyzed once all pullulan was consumed, after 8 and 14 days for the compost and soil enrichment, respectively. Images of the enrichments were taken using a Zeiss Axio microscope at 100× magnification.

In addition, a 250 mL flask was inoculated with 0.25 g compost combined with 0.25 g soil collected on the TUD campus and incubated at 75 °C in a water bath. The enrichment was monitored as described above.

### Monitoring degradation of pullulan by HPLC

Approximately 1 mL of culture broth was centrifuged for 10 min at 4 °C, 14 000 rcf, after which 667 μL of the supernatant was transferred to a clean screw cap vial. Trifluoroacetic acid (TFA) was added to reach a final concentration of 4 M TFA. The samples were incubated for 4 hours at 100 °C in a ThermoMixer with a ThermoTop (Eppendorf, ThermoMixer C). After a 15-min centrifugation step at 4 °C and 14 000 rcf, the supernatant was collected in a clean Eppendorf tube. The samples were measured using a Vanquish HPLC system (Thermo Scientific, Germany) with an Aminex HPX-87H separation column. A constant flow rate of 0.750 mL min^−1^ was maintained over a total run time of 45 minutes, using 1.5 mM phosphoric acid in Milli-Q water as the eluent. The column chamber temperature was kept at 50 °C and compounds were detected using an RI detector (ERC, RefractoMax 520). Data analysis was performed using Chromeleon 7 (Thermo Scientific, Germany).

### Whole metagenome sequencing

Whole metagenome sequencing of the starting biomass and enrichments was performed using BaseClear B.V. (Leiden, The Netherlands). Briefly, DNA was extracted using standard molecular biology kits from Zymo Research, and DNA concentration was confirmed using a NanoDrop spectrophotometer. Sequence libraries were generated using the Nextera XT DNA Library Preparation Kit. Paired-end sequence reads were generated using the Illumina NovaSeq PE150 system. The sequence generation was performed under accreditation according to the scope of BaseClear B.V. (L457; NEN-EN-ISO/IEC 17025). FASTQ read sequence files were generated using bcl2fastq version 2.20 (Illumina). Initial quality assessment was based on data passing the Illumina Chastity filtering. Subsequently, reads containing the PhiX control signal were removed using an in-house filtering protocol. In addition, reads containing (partial) adapters were clipped (up to a minimum read length of 50 bp). The second quality assessment was based on the remaining reads using the FASTQC quality control tool version 0.11.8. Metagenome assembly was performed using MEGAHIT 1.2.9. Contigs smaller than 1000 base pairs were removed from the final assembly. PROKKA was used to locate open reading frames (ORFs) on contigs and to translate the ORFs to protein sequences. These sequences were used as a reference database for the metaproteomic analysis. Taxonomic classification of ORFs was performed using the DIAMOND sequence aligner and the NCBI sequence reference database. A consensus lineage for every ORF was obtained by using the bit score approach from the top 25 alignments described earlier.^35,38^ Functional classification was performed as described below. In addition, a sequence alignment of the *CdaA* (UniProt: Q9WX32) and *AmyA* (UniProt: Q06307) genes of *Alicyclobacillus acidocaldarius* was performed against the metagenomics data of both the enrichment cultures and the inocula using the DIAMOND sequence aligner.

### Metaproteomics on microbial enrichments

The biomass of the enrichment cultures was collected in a clean LoBind Eppendorf tube (approx. 25 mg wet weight). Subsequently, 0.175 mL of TEAB resuspension buffer (50 mM TEAB, 1% (w/w) sodium deoxycholate, pH 8), 0.175 mL of the B-PER reagent (Bacterial Protein Extraction Reagent, Thermo Scientific) and 0.15 g of acid washed glass beads (150–212 μm) were added. The samples were homogenized using a bead beater for 1.5 min, followed by a 1-minute incubation on ice and a centrifugation for 2 min at 4 °C and 14 000 rcf. This cycle was repeated twice. Afterwards, the samples were subjected to an ultrasonic bath for 10 min and centrifuged for 15 min at 4 °C and 14 000 rcf. All centrifugation steps were conducted at 4 °C and 14 000 rcf unless stated otherwise. The supernatant was collected in a LoBind Eppendorf tube. Trichloroacetic acid (TCA) was added in a 1 : 4 ratio to the supernatant. The samples were vortexed and incubated for 30 min at 4 °C, followed by centrifugation for 15 min. The supernatant was discarded, and the protein pellet was washed with 200 μL of ice-cold acetone, vortexed and centrifuged for 15 min. The supernatant was removed and 100 μL of 6 M urea was added to the protein pellets. To re-dissolve the pellets, the samples were vortexed thoroughly and incubated at 37 °C and 300 rpm in a ThermoMixer with a ThermoTop (Eppendorf, ThermoMixer). If necessary, an additional 100 μL of 6 M urea was added to dissolve the pellets. The samples were reduced by incubating at 37 °C for 60 min after adding 30 μL of 10 mM dithiothreitol and subsequently alkylated for 30 minutes at RT in the dark by adding 30 μL of 20 mM iodoacetamide. The samples were diluted with 200 mM ammonium bicarbonate to achieve a urea concentration < 1 M. To 100 μL of the final sample volume, 5 μL of 0.1 μg μL^−1^ trypsin solution was added. After gentle shaking, the samples were incubated at 37 °C overnight. The samples were cleaned and concentrated using the Oasis HLB 96-well μElution Plate with 2 mg sorbent per well, 30 μm (Waters, UK). In short, the columns were conditioned with 750 μL MeOH, equilibrated with 2 × 500 μL MS-H_2_O and then loaded with the samples. Two washing steps with 350 μL 5% MeOH in MS-H_2_O were performed, followed by elution with 200 μL of 2% formic acid in 80% MeOH and 200 μL of 1 mM ammonium bicarbonate (ABC) in 80% MeOH. The eluates were collected in a LoBind Eppendorf tube. The samples were dried using a SpeedVac concentrator (Thermo Scientific) at 50 °C and stored at −20 °C until analysis by LC–MS. For LC–MS analysis, the dried samples were dissolved in 15 μL of 3% ACN plus 0.1% trifluoroacetic acid (TFA) in MS-H_2_O, incubated for 30 min at RT, and vortexed thoroughly. The samples were diluted to a final protein concentration of approximately 0.5 mg mL^−1^, estimated at 280 nm using a NanoDrop ND-1000 spectrophotometer (Thermo Scientific), before proteomic analysis.

Extracellular proteins from the secretome of the enrichment cultures were prepared as described in the following. Samples from the enrichment culture were centrifuged for 15 min and the supernatant was collected. Several protein precipitation methods were employed including TCA precipitation as described above: acetone precipitation, acetone/salt precipitation and filter assisted sample preparation (FASP). The latter three are described below. For acetone precipitation, a 250 μL sample was mixed with 1250 μL ice-cold acetone, vortexed and stored at −20 °C for 30 min. The samples were centrifuged for 15 min and the supernatant was removed. For acetone/salt precipitation, 50 μL of 3 M NaCl solution was added to a 250 μL sample. Subsequently, 1200 μL acetone (RT) was added and the samples were mixed gently. The samples were incubated for 30 min at RT and centrifuged for 15 min. The supernatant was carefully removed, and the protein pellet was washed with 400 μL acetone (RT). Another 15-minute centrifugation was performed, and the supernatant was removed. After protein precipitation, the protein pellet was re-dissolved in 100 μL of 6 M urea and processed as described above. For filter assisted sample preparation, 200 μL of 6 M urea was added to a 10 kDa Microcon filter (Merck-Millipore), followed by centrifugation for 30 min at 14 000 rcf and 20 °C. After discarding the flow-through, this step was repeated once. Unless stated otherwise, all centrifugations during FASP were conducted at 14 000 rcf and 20 °C. Either a 250 μL sample or a 4 × 500 μL sample was loaded onto the filter followed by centrifugation for 30 min. The samples were then reduced by adding 30 μL of 10 mM DTT and 70 μL of 200 mM ammonium bicarbonate. After vortexing and incubating at 37 °C for 60 min, 30 μL of 20 mM IAA was added to alkylate the samples. Following 30-min incubation in the dark at RT, the samples were centrifuged for 30 min. Subsequently, a wash and equilibration step was performed by first adding 100 μL of 6 M urea and centrifuging for 30 min, followed by adding 100 μL of 200 mM ammonium bicarbonate twice and centrifuging for 30 min. The filter was transferred to a clean collection tube and 5 μL of 0.1 μg μL^−1^ trypsin solution together with 95 μL of 200 mM ammonium bicarbonate was added to the filter. The filter was incubated overnight in a ThermoMixer with a ThermoTop at 37 °C. Sample collection was performed by centrifuging for 30 min. Subsequently, 150 μL of 200 mM ammonium bicarbonate was added to the filter, followed by centrifugation for 30 min. The samples were again centrifuged for 30 min after the addition of 150 μL of 10% acetonitrile (ACN) and 0.1% formic acid (FA) in MS-H_2_O. Finally, the samples were cleaned and concentrated using solid phase extraction, as described previously. The results from the different sample preparation methods applied to identify extracellular proteins were combined before further analysis for glycosyl hydrolase enzyme candidates.

Shotgun proteomics was performed on an EASY-nLC 1200 coupled with a Q Exactive Plus Orbitrap mass spectrometer (Thermo Scientific, Germany). Chromatographic separation was carried out on a 0.05 × 150 mm^2^ C18 column (Thermo Scientific, catalogue no. 164943), with mobile phase A consisting of 0.1% formic acid (FA) and 1% acetonitrile (ACN) in MS-H_2_O and mobile phase B consisting of 0.1% FA and 80% ACN in MS-H_2_O. The chromatographic profile included an initial 2-minute run with 5% B, followed by a linear gradient from 5% to 25% B over 90 min, then a gradient to 55% B over 60 min. The gradient was subsequently returned to 5% B within 3 min and equilibrated for an additional 20 min, all at a constant flow rate of 350 nL min^−1^. Sample injections of 2 μL were performed with blank runs between each sample. Electrospray ionization operated in the positive mode, and MS1 analysis was conducted at a resolution of 70 000, with an AGC target of 3e6 and a maximum injection time of 75 ms. Applying the data dependent acquisition mode, precursors for fragmentation were isolated using a 2.0 *m*/*z* window over the scan range of 385–1250 *m*/*z*. A normalized collision energy (NCE) was set at 28%. MS2 spectra were collected at a resolution of 17 500, an AGC target of 2e5 and a maximum injection time of 75 ms. The database search was performed using PEAKS Studio 10.5 (Bioinformatics Solutions Inc., Canada) with the sequence database obtained from whole metagenome sequencing experiments (described above) and the GPM cRAP contaminant database. Carbamidomethylation was considered a fixed modification, while oxidation and deamidation were included as variable modifications. Trypsin was used as the proteolytic enzyme, allowing a maximum of three missed cleavages. The parent mass error tolerance was set to 20.0 ppm and the fragment mass error to 0.02 Da. Finally, filtering criteria included a 1% false discovery rate (FDR) for peptide-spectrum matches and a requirement of at least 2 unique peptides for protein identification.

### Functional annotation pipeline

To facilitate the identification of the target glycoside hydrolase, a streamlined bioinformatics pipeline was established. For this, a Virtual Box (Oracle VM Virtual Box, version 7.0.2) was set up with Linux Mint 21 (Cinnamon 64 bit) and the pipeline was operated using Python 3.10.12. The functional annotation pipeline was initiated within the python module. The input metagenomics fasta file is first split into smaller files containing a maximum of 1000 proteins each. The program then loops through these files and performs an annotation using HMM sequence models by using InterProScan (version 5.59-91.0). InterProScan is run from within the python program using the ‘subprocess’ module. The split fasta files serve as an input, generating an output in the xml format. The precalculated match lookup service of InterProScan was disabled to run everything locally. After the annotation with InterProScan, a keyword search was performed to mine for sequences that contain elements matching the target functions. Lastly, the data were combined with the metaproteomics results (identified proteins and abundances).

### Expression of the enzyme candidate MMBJNONL_14124

Expression of the enzyme candidate MMBJNONL_14124 was carried out by trenzyme GmbH (Germany). In short, a codon-optimized gene sequence, including a C-terminal His-tag, was synthesized and cloned into the expression vector pTZ_E02_(1)_XX124. Protein expression was performed in the host strain *Rosetta2 (DE3)*. A 100 mL culture in FM medium was grown at 37 °C; during autoinduction, the temperature was shifted to 23 °C. The total cultivation lasted 43 h, reaching a final OD_600_ of 14.1 and yielding 3.0 g of biomass.

### Cell lysis

Approximately 1.5 g of biomass was resuspended in 7.5 ml of 50 mM potassium phosphate (pH 7.4), containing 1 mM MgCl_2_, a spatula tip of DNAse and one cOmplete™ mini Protease Inhibitor Cocktail tablet. The cells were lysed using Lysin Matrix B (MP Biomedicals) in a FastPrep-24 Classic instrument (MP Biomedicals) for 2 cycles of 45 s at 6.5 m s^−1^ with a 5-min break on ice in between. After centrifuging for 15 min at 14 000 rcf and 4 °C, the SN was filtered using a 0.22 μm filter. Purification of the His-tagged protein from the cell free extract (CFE) was performed on a Bio-Rad NGC Chromatography System using a 1 mL HisTrap FF crude column. The column was equilibrated with 5 column volumes (CVs) of 100% mobile phase A (50 mM potassium phosphate pH 7.4, 300 mM NaCl, 20 mM imidazole and 1 mM DTT), after which the CFE was loaded onto the column. A wash phase of 10 CVs with mobile phase A was performed, followed by a linear gradient to 100% mobile phase B (50 mM potassium phosphate (pH 7.4), 300 mM NaCl, 500 mM imidazole and 1 mM DTT) over 40 CVs, while collecting 0.5 mL fractions. Fractions containing the purified protein (A16–A35) were pooled and concentrated using an Amicon® Ultra Centrifugal Filter (MWCO: 10 kDa). The buffer was exchanged with 50 mM potassium phosphate buffer (pH 7.4) using a PD10 desalting column (GE Healthcare), according to the manufacturer's protocol. After eluting with 2.5 mL of 50 mM potassium phosphate buffer (pH 7.4), the sample was again concentrated to ∼0.5 mL.

### SDS-PAGE

Proteins were separated by SDS-PAGE on a 4–15% Mini-PROTEAN® TGX Stain-Free™ Protein Gel. As a reference, the Precision Plus Protein™ Unstained Protein Standards (Strep-tagged recombinant) were loaded onto the gel. A 15 μL sample was denatured in 14.5 μL 2× Laemmli buffer (65.8 mM Tris-HCl, pH 6.8, 2.1% SDS, 26.3% (w/v) glycerol, and 0.01% bromophenol blue) and 0.5 μL of 100 mM DTT at 95 °C for 5 min before loading onto the gel. Electrophoresis was performed at 120 V in 10× tris/glycine/SDS buffer until the dye front reached the bottom of the gel (∼30 min). Proteins were visualised using a ChemiDoc MP Imaging system from Bio-Rad with optimal auto exposure for 45 s.

### Activity assay confirming the pullulan hydrolytic activity of MMBJNONL_14124

Reactions with a final volume of 100 μL were prepared in 0.6 mL tubes, each containing 1 mg mL^−1^ pullulan, 5 mM CaCl_2_ and 100 mM sodium acetate (pH 4.5). Activity assays were performed first with the cell free extract and subsequently with the purified enzyme. The CFE was diluted five times in 125 mM sodium acetate (pH 4.5) and subsequently diluted three times in H_2_O. 50 μL of the diluted CFE was added to a 50 μL reaction mixture (final volume of 100 μL). 50 μL of the purified enzyme (∼1.5 mg mL^−1^ in 50 mM potassium phosphate (pH 7.4)) was diluted in 100 μL Optima LC/MS-grade H_2_O. Subsequently, 50 μL of this dilution was added to the 50 μL reaction mixture. The reaction mixtures were incubated overnight at 60 °C. Negative controls lacking either the cell free extract/enzyme or the substrate (pullulan) were included. The samples were washed and concentrated using a HyperSep^TM^ Hypercarb^TM^ SPE 96-well plate (25/1 mL plate, Thermo Scientific). Briefly, the columns were washed with 750 μL 10 mM ammonium bicarbonate (ABC) in 60% Optima LC/MS-grade acetonitrile (ACN) and equilibrated with 2 × 500 μL Optima LC/MS-grade H_2_O. Each sample was diluted to 200 μL and centrifuged at 14 000 rpm for 5 minutes to remove insoluble material. The samples were then applied to the PGC cartridges, washed twice with 200 μL H_2_O, and the carbohydrate mono- and oligosaccharide fraction was eluted with 300 μL of 10 mM ABC in 60% ACN. Eluates were collected in Eppendorf tubes, dried in a SpeedVac concentrator at 60 °C and re-dissolved in 50 μL H_2_O. For PGC-MS/MS analysis, 2 μL of this sample was injected to the LC–MS system.

### PGC-MS/MS analysis

Liquid chromatographic separation coupled with high resolution mass spectrometric detection was conducted using an Acquity Ultra Performance Liquid Chromatograph (Waters, UK) coupled with a Q Exactive™ Focus Hybrid Quadrupole-Orbitrap™ mass spectrometer (Thermo Scientific, Germany). Chromatographic separation was performed on a 100 × 1 mm^2^ Hypercarb™ Porous Graphitic Carbon HPLC column (Thermo Scientific, no. 35005-101030) using a constant flow rate of 100 μL min^−1^ and an electrical grounding between the ESI source and the column inlet. Mobile phase A consisted of 0.1% formic acid (FA) in Optima LC/MS-grade H_2_O and mobile phase B consisted of 0.1% FA in Optima LC/MS-grade acetonitrile. The column was equilibrated with 1% B for 5.1 min, followed by a linear gradient to 40% B over 4.9 min. The system was returned to the initial conditions and equilibrated for another 4 min. 2 μL injections were performed, analysing each sample in duplicate with a wash between each sample. MS1 spectra were acquired in positive mode at a resolution of 70 000 over the scan range of 150–1250 *m*/*z*. The automatic gain control (AGC) was set to 1e6 and the maximum injection time (IT) to auto. Precursors for product hexose mono- and oligosaccharides were included for continuous isolation and fragmentation in PRM mode. The MS2 spectra were acquired at a resolution of 35 000, with the AGC set at 2e5 and the maximum IT at auto. Fragmentation occurred with a 25% normalized collision energy. Raw data were processed using Xcalibur 4.1 (Thermo Fisher Scientific, Germany). Calibration of the mass spectrometer was performed using the Pierce™ LTQ ESI negative ion calibration solution (Thermo Fisher Scientific, Germany).

## Results

### Enrichment on pullulan as the sole carbon source

**Fig. 1:**
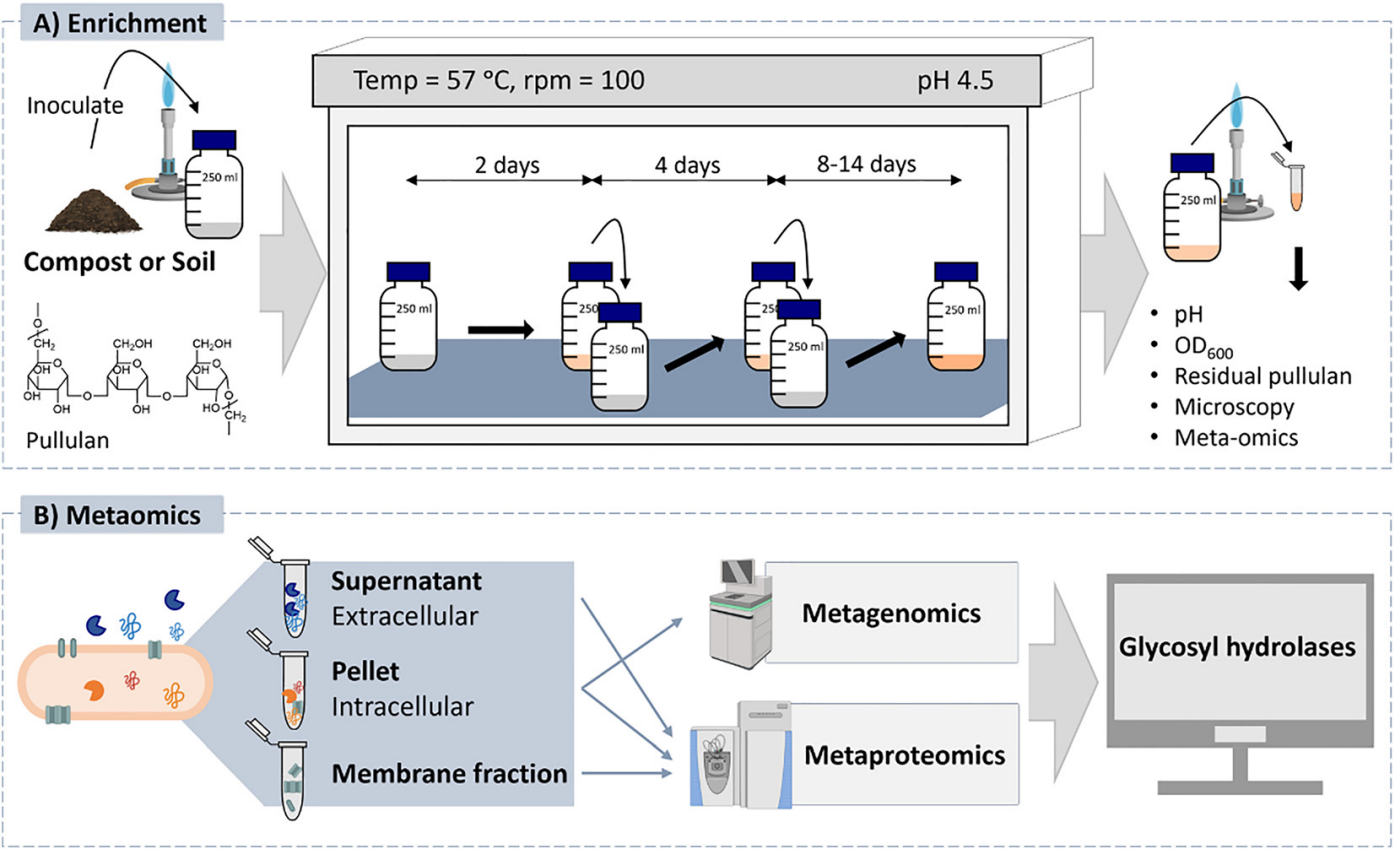
The employed workflow for the discovery of novel glycoside hydrolases from microbial enrichments. (A) Enrichment cultures at 57 °C and pH 4.5 were established by inoculating either with compost (elevated temperature microbial source) or soil (temperature source control) using pullulan as the sole carbon substrate. In addition, the growth medium without the inoculum served as the control for cross contamination. The pH, OD_600_ and residual pullulan were monitored daily. The enrichments were studied by microscopy and once all pullulan was depleted the biomass and the secretome were analysed by metagenomics and metaproteomics. (B) The enrichment biomass was subjected to whole metagenome sequencing. Additionally, metaproteomics analysis was performed on both the cell biomass and supernatant fractions (secretome). While not utilized in this study, future metaproteomic investigations could include a separate analysis of the membrane fraction, offering deeper insights into substrate degradation and uptake pathways, by identifying dedicated transporters. The subsequent functional annotation pipeline was tailored to identify potential glycoside hydrolase candidates.

To enrich for microbes capable of degrading pullulan under thermophilic (57 °C) and acidic (pH 4.5) conditions, pullulan was supplied as the sole carbon source. The impact of the inoculum on the enrichment of the microbial communities, and therefore the enzymes obtained, was evaluated by establishing two enrichment cultures. The first, referred to as the ‘compost enrichment’, was inoculated with organic matter from a compost pile. This microbiome was already exposed to elevated temperatures during composting. The second enrichment, called ‘soil enrichment’, was inoculated with soil from the bank of a pond, which was exposed to environmental temperatures (5–20 °C). A control culture without the inoculum was included, and all experiments were conducted aseptically to prevent cross-contamination (Fig. 1). In pursuit of enzymes capable of functioning at even higher temperatures, one additional enrichment was conducted at 75 °C.

**Fig. 2:**
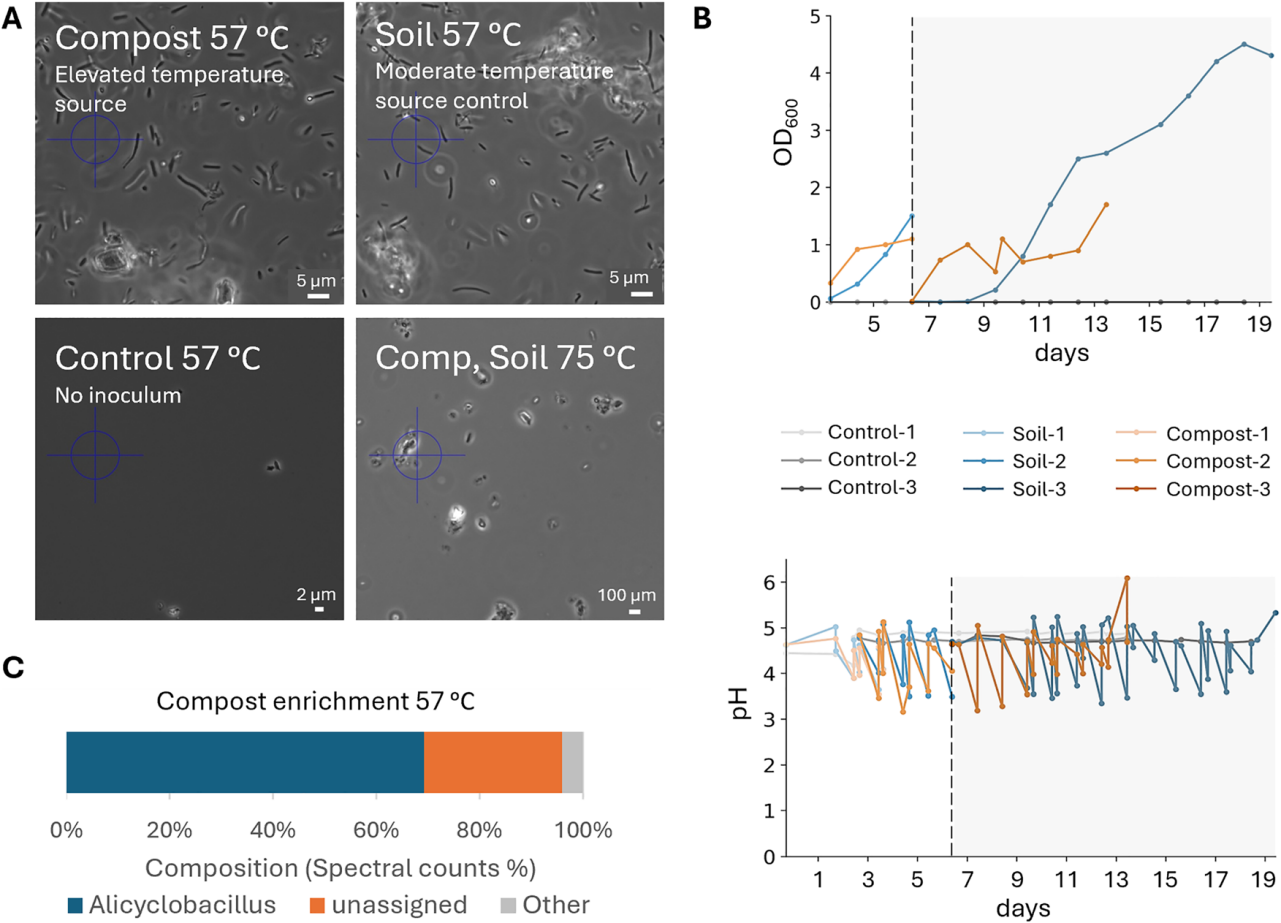
(A) Microscopic images of the enrichment cultures at 100× magnification. Compost (elevated temperature microbial source): inoculated with organic matter of a compost pile. Soil (temperature source control): inoculated with sediments from a pond. Control: no inoculum. Comp, Soil 75 °C: inoculated with both compost and soil and incubated at 75 °C. All images were taken two days after inoculation. (B) pH and OD_600_ measurements during the compost, soil and control enrichment cultures. Grey area: the period after the second transfer to fresh medium. (C) Taxonomic composition (genus level) based on spectral counts (metaproteomics) of the ‘Compost’ enrichment biomass, normalized to 100%. Approximately 70% of the counts could be assigned to the genus *Alicyclobacillus*. The legend shows genera with >10 spectral counts (for full annotation see Fig. S4).

Two days after inoculation, microbial growth was confirmed by microscopy and the increase in OD_600_ in both the ‘compost’ and ‘soil’ enrichments, while absent in the control (Fig. 2A and B). In addition, no growth was observed for the enrichment at higher temperature (75 °C), which was subsequently terminated. Both the ‘compost’ and ‘soil’ enrichments were observed to acidify over time (Fig. 2B). This required daily pH adjustments with KOH, since no organic buffer was used to prevent it from becoming a substrate for the microorganisms. Assuming the following biomass growth stoichiometry, acidification was likely the result of ammonium uptake (see the SI). Moreover, HPLC-UV analysis using an Aminex organic acid analysis column showed no additional peaks indicative of organic acids, excluding the possibility of acid production during the enrichment (SI, Fig. S1). To confirm pullulan utilization, the residual pullulan in the medium was hydrolysed to glycose and analysed by HPLC. The glucose levels decreased over time in the ‘compost’ and ‘soil’ enrichments, but remained constant in the control, verifying the consumption of pullulan by microorganisms (SI, Fig. S2).1 × C_6_H_12_O_6_ + 0.656 × NH_4_^+^ + 2.56 × O_2_ → 3.28 × CH_1.8_N_0.2_O_0.5_ + 2.72 × CO_2_ + 4.03 × H_2_O + 0.6 × H^+^

### *Alicyclobacillus* identified as the dominant genus in both enrichments

The inocula and the biomass from the enrichments were analyzed using whole metagenome sequencing and metaproteomics. Interestingly, the compost inoculum was found to contain a complex spectrum of microorganisms based on a taxonomic classification of the metagenomics data. In total, the classification of the obtained open reading frames (ORFs) yielded more than 100 different genera, including *Geofilum*, *Capillibacterium*, *Methanoculleus*, and *Methanosarcina* (SI, Fig. S3). Over 80% of the identified genes could not be classified using this method, pointing to a significant proportion of yet undescribed microbes. This finding highlights the vast diversity and novelty of microorganisms in the inoculum. After enrichment on pullulan at 57 °C and pH 4.5, the complexity in both enrichments reduced to one dominant genus, namely *Alicyclobacillus* (Fig. 2C and Fig. S4, SI), indicating that, despite the different origins of the inocula, the imposed conditions could select for the same microbe.

### Identification of new glycoside hydrolases from *Alicyclobacillus*

**Fig. 3:**
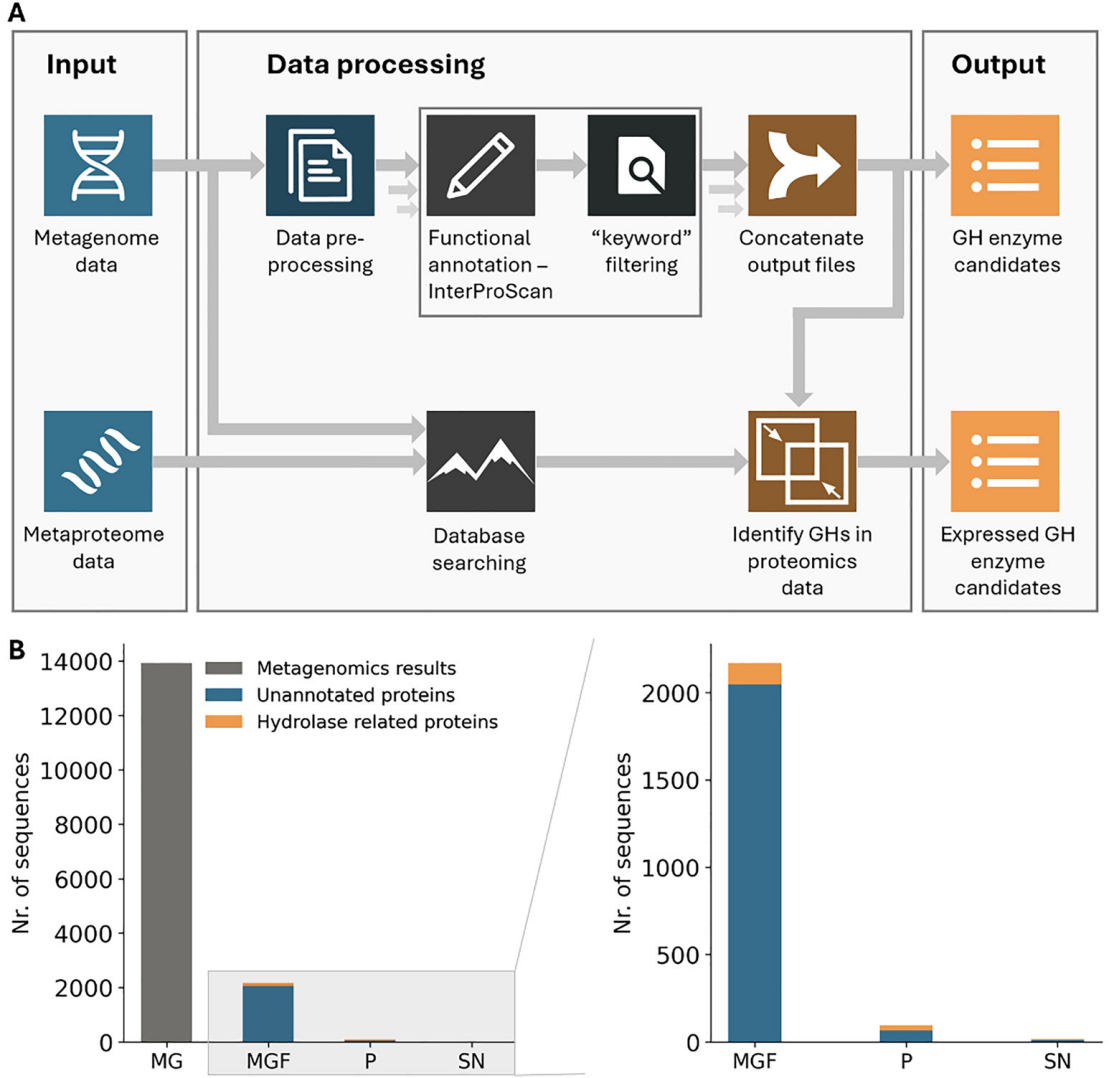
(A) Schematic overview of the employed bioinformatics pipeline. Functional annotation of the whole metagenome sequencing data was performed using InterProScan. Functional entries containing hydrolase-related keywords (see Table S1) were flagged as potential glycoside hydrolases. The annotated database was subsequently employed to analyse the metaproteomics data by database search, which ultimately identified a focused number of glycoside hydrolase (GH) candidates that were confirmed to be expressed. (B) The number of pullulan-degrading enzyme candidates obtained from the metaproteomics approach is compared to the total number of sequences obtained from the whole metagenome sequencing data. While metagenomic datasets typically encompass thousands of genes, combining these data with comprehensive functional classification and metaproteomics experiments on both cell pellet and supernatant fractions narrows the focus to a specific set of glycoside hydrolase candidates. In the case of pullulan, this approach also confirmed a two-step degradation process occurring both extracellularly and intracellularly. MG: number of genes obtained from the ‘compost’ enrichment by whole metagenome sequencing, MGF: number of GH candidates based on comprehensive functional classification of metagenomics data, P: number of GH candidates based on metaproteomics on the cell pellet, SN: number of GH candidates based on metaproteomics on the supernatant fraction (secreted proteins).

To identify potential glycoside hydrolases that enable *Alicyclobacillus* to grow efficiently on pullulan, we established a pipeline that combines metagenomic functional annotation with metaproteomic expression and localization data (Fig. 3A). In short, the pipeline annotates proteins using the InterPro database, with a local InterProScan installation, providing an overview of protein families, domains, and sites for each gene. The annotated data were subjected to a keyword search to identify potential enzymes related to hydrolytic activity and pullulan degradation. Keywords included “family 13,” “GH13,” “family 57,” “GH57,” “amylase,” “glycosidase,” “glycoside hydrolase,” and “pullulan”. Unannotated genes were also retained in the final list to allow identification of completely novel sequences. The subsequent integration of metaproteomics data provided information on the expression levels and localization of these proteins.

Whole metagenomic sequencing of the ‘compost’ enrichment yielded ∼14 000 genes. Applying the functional annotation and keyword filtering, this could be narrowed down to ∼2000 genes. However, validating the activity of each gene would remain a costly and time-consuming process. Advantageously, integrating metaproteomics data reveals enzymes that are actively expressed. Because pullulan degradation is essential for growth, relevant enzymes were expected to be highly abundant. Moreover, protein localization was inferred by analyzing supernatant and biomass pellet fractions separately, thereby distinguishing secreted enzymes from intracellular enzymes. This particularly supports the identification of enzymes that are involved in the degradation of the carbon source before cellular uptake.^39^ Remarkably, for the compost enrichment, integrating the metaproteomics data reduced the number of enzyme candidates to 96 intracellular and 17 extracellular (Table 1). Therefore, employing metaproteomics significantly narrowed down the pool of target enzyme candidates. This greatly streamlines subsequent functional and structural investigations, as well as biocatalytic testing after recombinant expression.

**Table 1:** Identified pullulan degrading enzyme candidates in the supernatant (secretome) of the compost enrichment using metaproteomics. InterProScan annotations: IPR06047 – glycoside hydrolase, family 13, catalytic domain; IPR004185 – glycoside hydrolase, family 13, N-terminal Ig-like domain; IPR017853 – glycoside hydrolase superfamily; IPR015020 – Rv2525c-like, glycoside hydrolase-like domain; IPR015955 – lactate dehydrogenase/glycoside hydrolase, family 4, C-terminal (homologous superfamily). The bars show the spectral count based abundance of the identified proteins in the supernatant fraction (secreted proteins)

Protein group	Accession no.	MW	InterPro	Spectral counts (abundance)
Glycoside hydrolase domain	MMBJNONL_14124	140 550	IPR006047, IPR004185, IPR017853	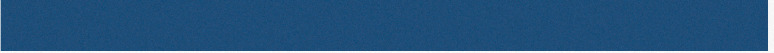
	MMBJNONL_07072	91 271	IPR006047, IPR004185, IPR017853	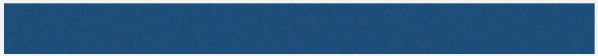
Glycoside hydrolase superfamily	MMBJNONL_03000	75 891	IPR015020, IPR017853	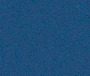
	MMBJNONL_05960	58 192	IPR017853	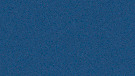
Lactate dehydrogenase/glycoside hydrolase superfamily	MMBJNONL_09916	33 232	IPR015955	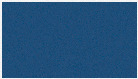
	MMBJNONL_07048	33 418	IPR015955	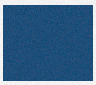
Proteins without any functional annotation by InterProScan	MMBJNONL_05225	21 160	—	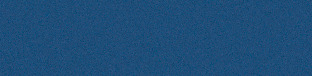
	MMBJNONL_03006	18 307	—	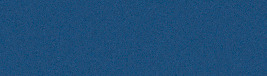
	MMBJNONL_05223	27 924	—	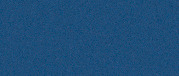
	MMBJNONL_09075	32 173	—	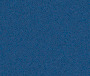
	MMBJNONL_01486	47 258	—	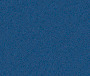
	MMBJNONL_05876	18 086	—	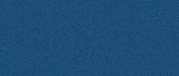
	MMBJNONL_03029	18 064	—	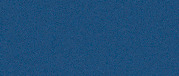
	MMBJNONL_02580	15 965	—	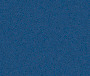
	MMBJNONL_02999	13 289	—	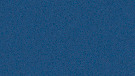
	MMBJNONL_07823	21 122	—	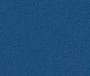
	MMBJNONL_01632	28 355	—	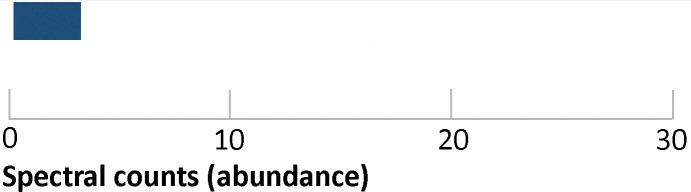

From the 17 extracellular candidates, six were annotated using InterPro, of which two (MMBJNONL_14124 and MMBJNONL_07072) were identified as containing a domain from glycoside hydrolase family 13 (GH13). These are the most likely candidates possessing pullulan degrading activity, since this family includes enzymes such as alpha-amylases and neopullulanases. Some expressed proteins lacked functional annotations and would also require further activity testing. The InterProScan annotation of the final extracellular pullulan-degrading enzyme candidates was further validated using dbCAN3^40^ (see Table S2, Fig. S6 and the SI). Both GH13 candidates were confirmed, and MMBJNONL_05960 was further classified by dbCAN3 as belonging to glycoside hydrolase family 53. Finally, MMBJNONL_09916 and MMBJNONL_07048 were annotated using InterProScan, but not recognized by dbCAN3. Both were assigned the domain IPR015955 and annotated as “lactate dehydrogenase/glycoside hydrolase”. If these enzymes primarily function as lactate dehydrogenases, it might explain their absence in the dbCAN3 annotation. Overall, the annotation results are highly consistent between the two tools and support the hypothesis that MMBJNONL_14124 and MMBJNONL_07072 are likely responsible for pullulan degradation.

**Fig. 4:**
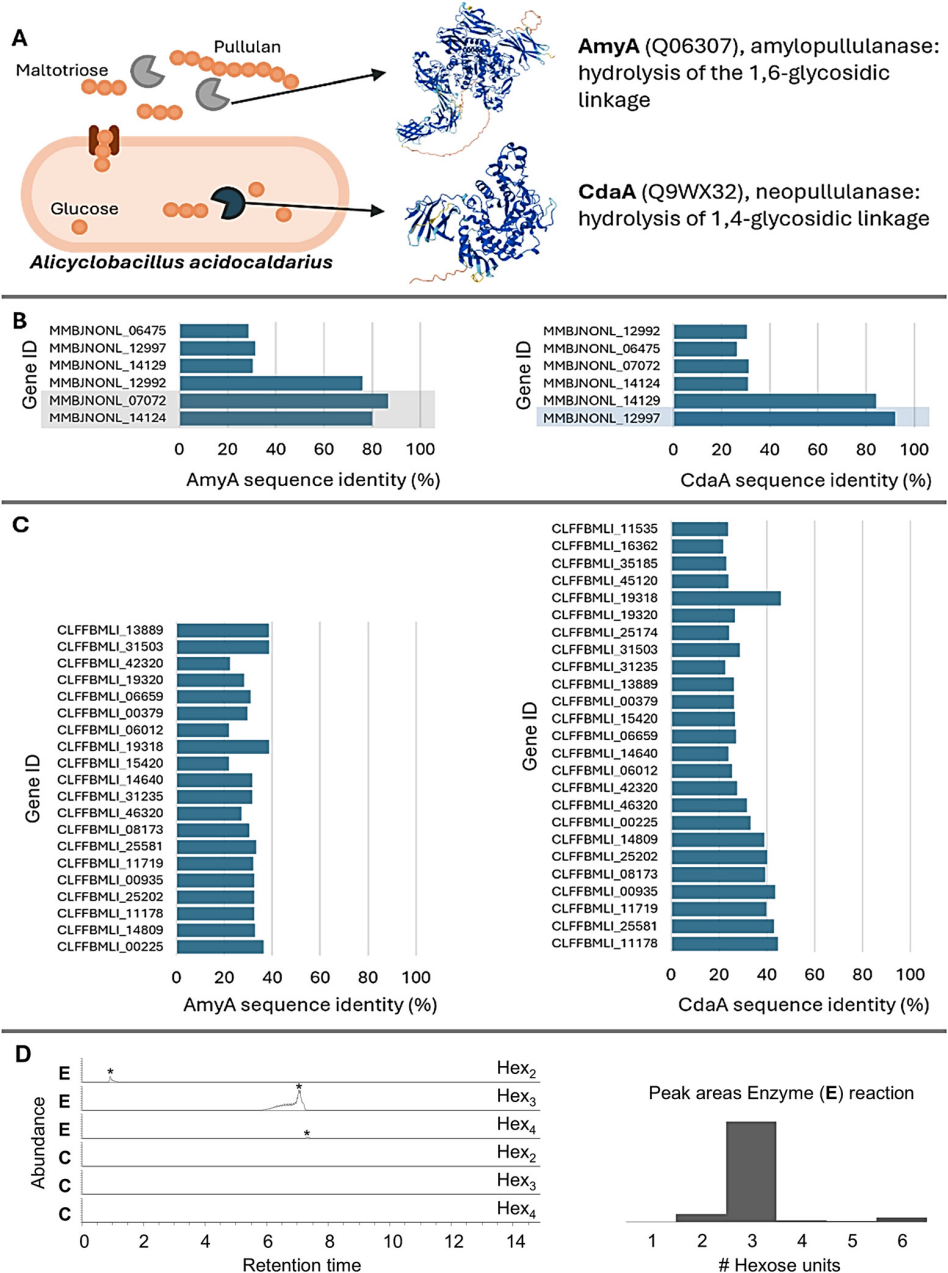
(A) The proposed pullulan degrading pathway in *Alicyclobacillus* based on the identified enzymes in this study and recent work performed by Matzke *et al.* and the enzyme activity assay performed in this study. AmyA hydrolyses the 1,6-glycosidic linkage of pullulan extracellularly. The maltotriose units are then imported, after which CdaA hydrolyses the 1,4-glycolytic linkage to form glucose, which can enter the glycolytic pathway. (B) Sequence alignment of the AmyA (Q06307) and CdaA (Q9WX32) genes against the metagenomics data of the compost enrichment culture. Genes showing high sequence identity and those that were identified with the glycoside hydrolase bioinformatics pipeline are highlighted in grey (extracellular) or in blue (intracellular). The identified genes with high sequence identity (MMBJNONL_12992, MBJNONL_07072, MMBJNONL_14124, MMBJNONL_12997 and MMBJNONL_14129) were not identified in the metagenomics data of the inoculum, which demonstrates the effectiveness of performing microbial enrichment combined with metaproteomics. (C) Sequence alignment of the AmyA (Q06307) and CdaA (Q9WX32) genes against the metagenomics data of the compost inoculum. (D) Enzyme activity assay using purified MMBJNONL_14124, which was recombinantly expressed in *E. coli*. The release of hexose oligomers from pullulan after incubation with MMBJNONL_14124 at 60 °C and pH 4.5 was monitored using PGC chromatographic separation and high resolution mass spectrometry detection. The left traces show the extracted ion chromatograms for Hex_2_, Hex_3_, and Hex_4_ units (*), where the upper three traces show the release after incubation with MMBJNONL_14124 (enzyme reaction, E) and the lower three traces show the control reaction where no enzyme was present (control reaction, C). The control reaction (buffer and pullulan) showed only trace amounts of the respective sugar oligomers. The *y*-axis of each EIC was scaled to NL: 7.50E6. The right histogram shows the peak area distribution for the different hexose oligomer products (Hex_1_ to Hex_6_), where Hex_3_ products dominated.

Matzke and co-workers previously identified two pullulan-degrading enzymes in *Alicyclobacillus*: the cytoplasmic protein cyclomaltodextrinase (CdaA), which hydrolyzes 1,4-linkages of pullulan to form panose, and amylopullulanase (AmyA), which hydrolyzes 1,6-linkages of pullulan to form maltotriose.^41^ AmyA likely hydrolyses pullulan into maltotriose extracellularly, which can be transported over the membrane and is further degraded by CdaA (Fig. 4A). A sequence alignment of AmyA and CdaA against the metagenomics data of the ‘compost’ enrichment resulted in six matches, with the best showing only 80% sequence identity (Fig. 4B). Moreover, the matches with high sequence identity to AmyA (MMBJNONL_07072 and MMBJNONL_14124, Fig. 4B) were identified as extracellular by the enzyme discovery approach, while the best match with CdaA (MMBJNONL_12997 in Fig. 4B) was classified as intracellular. These cellular localizations were further supported by SignalP-6.0^42^ and DeepLocPro^43^ predictions (SI, Table S1). Besides the 80% sequence identity, the same domains were identified at similar locations, being CBM34 and GH13_39 for MMBJNONL_07072, MMBJNONL_14124 and AmyA and GH13_20 for MMBJNONL_12997 and CdaA (SI, Fig. S6). Pullulan degrading enzymes are known to contain four conserved sequence regions,^44,45^ all of which have been identified in MMBJNONL_12997, while three have been identified in MMBJNONL_07072 and MMBJNONL_14124 (SI, Fig. S7 and S8), further supporting the pullulan degrading activity. Interestingly, sequence alignment of the AmyA and CdaA genes against the metagenomics data of the compost inoculum yielded matches with only ∼45% sequence identity, while the enzymes identified through the employed enrichment metaproteomics approach were not detected at all (Fig. 4). Although deeper genomic sequencing might detect these enzymes, this underscores the effectiveness of combining microbial enrichment with metaproteomics.

To validate the results of our established meta-omics approach and the associated functional predictions, the top extracellular pullulan-degrading enzyme candidate MMBJNONL_14124 was recombinantly expressed in *E. coli* with a C-terminal His-tag and purified by affinity chromatography. The purified enzyme was subsequently incubated with 1 mg mL^−1^ pullulan at 60 °C and pH 4.5, and pullulan hydrolysis was monitored using high-resolution tandem mass spectrometry. Thus, we observed a predominant release of triose oligomers (Fig. 4D), which aligned with the cleavage of α-1,6-glycosidic bonds and the release of maltotriose units from pullulan. This confirms the AmyA-like pullulan-hydrolytic activity of MMBJNONL_14124 under elevated temperature and acidic pH conditions.

## Discussion and conclusions

Advancements in next-generation sequencing have opened up new avenues for the discovery of novel enzymes from microbial sources.^28^ However, a purely sequence-based approach is time-consuming and commonly overlooks enzymes with new sequences, and the alternative function-based approach is costly and requires the availability of high-throughput activity assays.^2^ Therefore, methods that narrow down the pool of potential enzyme candidates without losing the ability to find completely novel enzymes are of great advantage. The combined application of enrichments with multi-omics allows us to search for completely novel enzymes operating under defined conditions. We exemplified this approach by searching for new pullulan-degrading enzymes capable of operating at elevated temperatures and acidic pH from two complex microbial sources (*i.e.* organic matter from compost and soil from a pond). The enrichment used pullulan as the sole carbon source, ensuring that only microorganisms with pullulan-degrading and utilizing capabilities would thrive. In addition to selecting for a desired function, this process allows customization of conditions to match industrial requirements, such as temperature, pH, and salinity. Nevertheless, the enrichment approach is limited to enzymes that are required for cellular growth or survival, such as enzymes involved in carbon or nitrogen source utilization. Following enrichment, the biomass can be analyzed by whole metagenome sequencing and metaproteomics. Whole metagenome sequencing alone identified approximately 14 000 genes in the compost enrichment, which was narrowed down to around 2000 candidates after functional classification. Integrating metaproteomics data further allowed us to focus on actively expressed enzymes and their specific locations, such as intracellular, membrane-bound, or extracellular. For the compost enrichment, this approach reduced the number of enzyme candidates to 96 intracellular and only 17 secreted enzymes.

Nevertheless, the presence of dead and consequently lysed cells can result in the identification of intracellular proteins in the supernatant. To assess the amount of lysed cells, we compared the identified proteins in the supernatant with the 50 most abundant proteins in the biomass pellet (determined by spectral counts). 18 of these proteins were not at all identified in the secretome, and a significant cytoplasmic contamination is therefore unlikely. In addition, the supernatant showed traces of only a few of the enzymes of the abundant glycolytic pathway (glyceraldehyde-3-phosphate dehydrogenase, phosphoglycerate mutase and glucose-6-phosphate isomerase), which further confirmed that there was no significant cell lysis taking place. Finally, some proteins (*e.g.* membrane proteins) are difficult to detect by conventional proteomics approaches and might be missed without additional optimisation of the sample preparation.^46^ To complement experimental data, we used SignalP-6.0^42^ and DeepLocPro^43^ to predict the cellular localization of the secretome. Of these proteins, 27% were predicted to contain a signal peptide, and 18% contained a signal peptide according to SignalP-6.0 and were predicted to be extracellular by DeepLocPro (SI). Signal peptides not necessarily imply secretion, proteins may be membrane bound or, in the case of Gram-positive bacteria, be retained in the periplasm. Similarly, proteins without a signal peptide can still end up in the secretome.^42^ Nevertheless, these predictive tools are useful in validating whether the final pullulan degrading enzyme candidates are indeed extracellular (SI, Table S1).

Despite *Alicyclobacillus* being identified as the dominant genus, other coexisting organisms, though present at lower abundance, were identified. Therefore, it cannot be excluded that these organisms are also involved in hydrolyzing pullulan, which can subsequently be consumed by multiple microbes. Intriguingly, a third enrichment was conducted at 75 °C, but no growth was observed. A review by Kahar *et al.* highlights diverse pullulan-degrading enzymes identified from microbial sources, demonstrating activity at high temperature and low pH.^13^ For instance, pullulanases from organisms like *Fervidobacterium nodosum* Rt17-B1 (an optimal temperature, *T*_opt_, of 80 °C and an optimal pH, pH_opt_, of 5), *Desulfurococcus mucosus* DSM 2162T (*T*_opt_ = 85 °C, pH_opt_ = 5), and *Thermoanaerobacter* sp. B6A (*T*_opt_ = 75 °C, pH_opt_ = 5) are some examples. However, enzyme activity at 75 °C and pH 4.5 does not guarantee that the organism can grow under these conditions. For instance, for *Fervidobacterium nodosum*, the growth range is restricted to pH 6.0–8.0. Moreover, this bacterium, as well as *Desulfurococcus mucosus* and *Thermoanaerobacter*, are obligate anaerobes, preventing growth under the conditions employed in this study.^47–49^

One enrichment was inoculated with compost (“compost” enrichment), which typically has an elevated temperature and is expected to host thermotolerant microbes. To assess whether such conditions are essential, a second enrichment was inoculated using soil from a pond (‘soil’ enrichment). Despite the complex and diverse starting communities, *Alicyclobacillus* dominated in both cases. This bacterium was first isolated from an acidic thermal environment in the Yellowstone national park by Darland and Brock.^50^ It is a spore-forming, rod-shaped bacterium that typically grows at temperatures of 45–70 °C (optimum 60–65 °C) and pH values of 2–6 (optimum 3–4), closely matching the applied enrichment conditions. Cross-contamination between the enrichments is unlikely, as no growth was observed in the control experiment. Thus, similar organisms thrived under identical enrichment conditions regardless of the inoculum's initial temperature. The similarity may also be attributed to the proximity of the sampling sites (∼20 km) and their shared soil environment. Both type I and II pullulanases commonly degrade starch, a natural plant polymer found in soil, which may support the growth of similar organisms.

Although previous studies identified two pullulan-degrading enzymes in *Alicyclobacillus*, AmyA and CdaA, our enrichment metaproteomics approach revealed distinct enzyme sequences with potentially altered properties (Fig. 4). The four conserved sequence regions of alpha-amylases^44,45^ were identified in the enzyme candidates, along with the same glycosyl hydrolase domains found in AmyA and CdaA. These similarities support the hypothesis that the enzymes exhibit pullulan degrading activity. The identified enzymes were not detected in the whole metagenome sequencing data of the inoculum, highlighting the effectiveness of our approach. Metaproteomics confirmed that the putative AmyA is extracellular, while the putative CdaA is intracellular. This suggests that AmyA hydrolyzes pullulan into maltotriose units extracellularly, which are then imported into the cell and further broken down by CdaA into glucose. Supporting this, we also detected the expression of a maltose/maltodextrin-binding periplasmic protein and the maltose/maltodextrin transport system MalG. This aligns with previous findings of a high-affinity, binding-protein-dependent ABC transport system specific for maltose and maltodextrins in *Alicyclobacillus*.^51^

Finally, the pullulan-degrading activity of the extracellular enzyme MMBJNONL_14124 was confirmed by recombinant expression followed by activity testing at 60 °C and pH 4.5. High-resolution mass spectrometry confirmed the efficient release of predominantly hexotrioses, consistent with the predicted cleavage of the α-1,6-glycosidic bonds and the release of maltotriose units. Nevertheless, some larger and smaller oligomers were also detected, which may arise from some promiscuous activity under the tested conditions.

In summary, we demonstrated the combination of a microbial enrichment and metaproteomics for discovering novel microbial glycoside hydrolases, as exemplified by a pullulan enrichment from two different microbial sources. The integration of metaproteomics significantly accelerated enzyme identification in these complex environments, reducing the number of genes requiring subsequent biochemical validation.

## Author contributions

JvE, MvL, GvK and MP conceived and designed the project. JvE and SvS performed the experiments. GvK and MP provided critical input to the experiments and/or protocols. JvE, SvS and MP conducted the data analysis. JvE and MP generated the figures and wrote the original draft. All authors contributed to reviewing and editing the manuscript.

## Conflicts of interest

There are no conflicts to declare.

## Supplementary Material

CB-OLF-D5CB00049A-s001

CB-OLF-D5CB00049A-s002

## Data Availability

Due to legal confidentiality requirements, the genomic and mass spectrometric raw data cannot be made publicly available, but may be provided by the corresponding author upon reasonable request. Supplementary information (SI) is available. See DOI: https://doi.org/10.1039/d5cb00049a.
