# Supplementary material for: Discovery of microbial glycoside hydrolases via enrichment and metaproteomics
Source: RSC Chem Biol. 2025 Sep 25;6(11):1809–21. doi: 10.1039/d5cb00049a (PMC12510369; doi:10.1039/d5cb00049a)
Supplement: CB-006-D5CB00049A-s001 [file CB-006-D5CB00049A-s001.pdf]

Supplementary Information (SI) for RSC Chemical Biology.
This journal is © The Royal Society of Chemistry 2025



## Supplementary information material to:

# Discovery of novel glycoside hydrolases via microbial enrichment and metaproteomics

**Jitske M. van Ede[1], Suzanne van der Steen[1], Geert M. van der Kraan[2], Mark C.M. van Loosdrecht[1] and Martin Pabst[1,*]**

[1]Department of Biotechnology, Delft University of Technology, Delft, The Netherlands

[2]Genencor International B.V., International Flavors & Fragrances, Oegstgeest, The Netherlands

*Contact: m.pabst@tudelft.nl

## Table of Contents

S1. Culture acidification ..... 2

S2. HPLC chromatograms showing the Pullulan depletion ..... 3

S3. Taxonomic composition of the enrichment cultures and the inoculum ..... 5

S4. Optimal temperature and pH of pullulan degrading enzymes ..... 7

S5. Predicting cellular localization using SignalP and DeepLocPro ..... 8

S6. dbCAN3 annotation of the final pullulan degrading enzyme candidates ..... 9

S7. Multiple sequence alignment ..... 10

S8. Expression and purification of MMBJNONL_14124 in *Rosetta2 DE3* ..... 13

S9. PGC-MS/MS based pullulan degrading activity assay ..... 15

## S1. Culture acidification

Over time, the pH of both the 'compost' and 'soil' enrichments decreased, necessitating daily adjustments with KOH (Figure 2B). This acidification was likely a result of ammonium uptake for biomass growth. Initially, each enrichment contained 0.5 g of pullulan (which equals 3.09 mmol of glucose). Throughout the enrichment process, approximately 2.61 ml of 0.2 M KOH (0.52 mmol KOH) was added to maintain the pH at 4.5. Thus, for every mole of glucose consumed, 0.17 mol of KOH was needed to neutralize the produced $H^+$, indicating that 0.17 mol of $H^+$ was generated during growth.
The following biomass growth stoichiometry (normalized to the consumption of 1 mol glucose) is assumed:

$$1 * \mathrm{C_6H_{12}O_6} + 0.656 * \mathrm{NH_4^+} + 2.56 * \mathrm{O_2} \rightarrow 3.28 * \mathrm{CH_{1.8}N_{0.2}O_{0.5}} + 2.72 * \mathrm{CO_2} + 4.03 * \mathrm{H_2O} + 0.66 * H^+$$

According to this equation, the consumption of 1 mol of glucose could produce up to 0.66 mol of $H^+$. The observed acidification in the culture falls within the theoretically possible range of acidification.

## S2. HPLC chromatograms showing the Pullulan depletion

To confirm that the microorganisms were growing on pullulan, the pullulan depletion was monitored over time by HPLC, both before (Figure S1) and after complete hydrolysis (Figure S2).

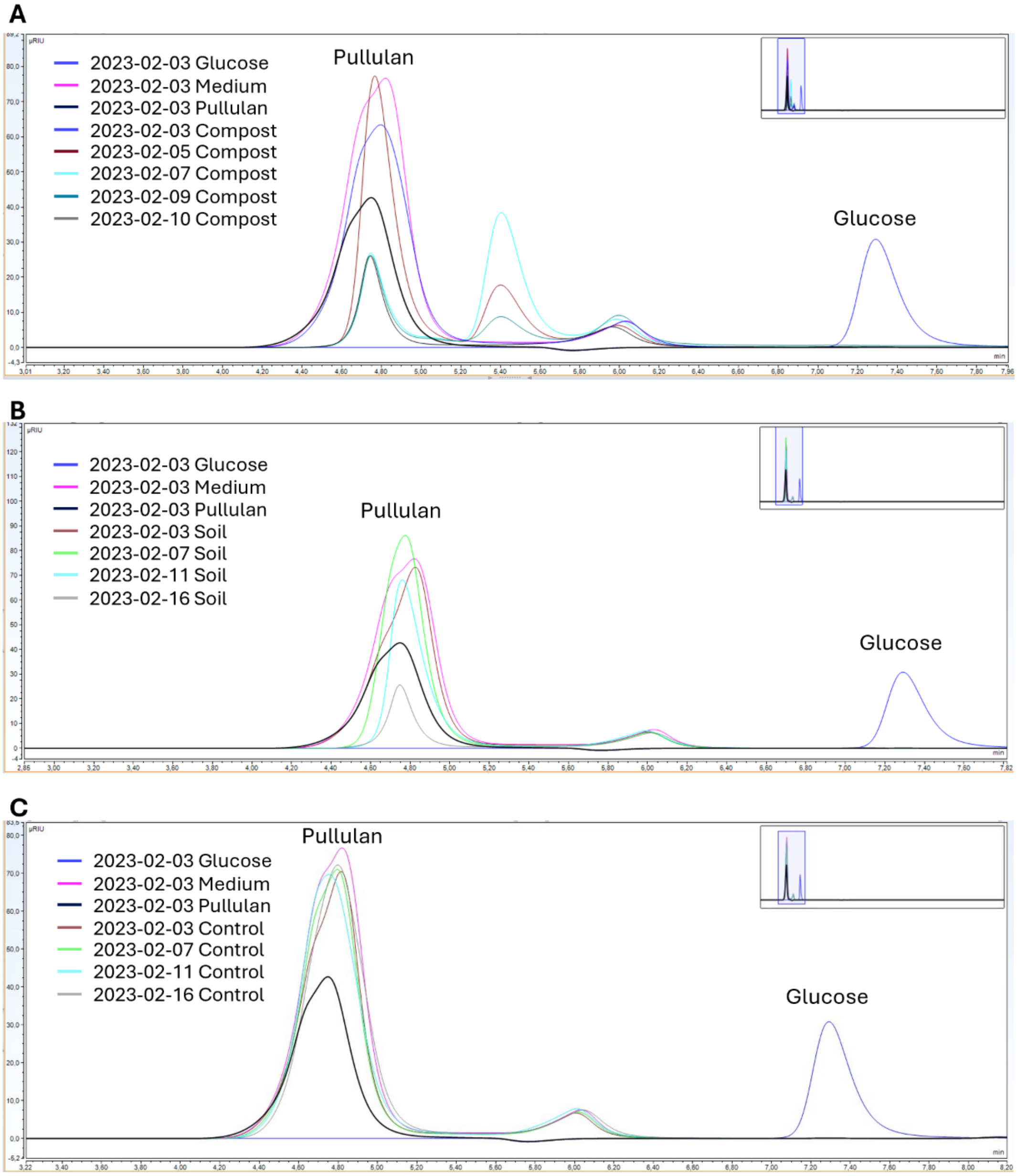


***Figure S2.1.*** Pullulan depletion. Overlayed HPLC chromatograms of the analysed enrichment cultures to follow the pullulan depletion over time. Whole broth samples were centrifuged after which the supernatant was analysed. HPLC conditions are described in the method section. **A)** Compost enrichment (elevated temperature microbial source): inoculated with organic matter of a compost pile. **B)** Soil enrichment (temperature source control): inoculated with sediments from a cold water pond. **C)** Control: No inoculum.

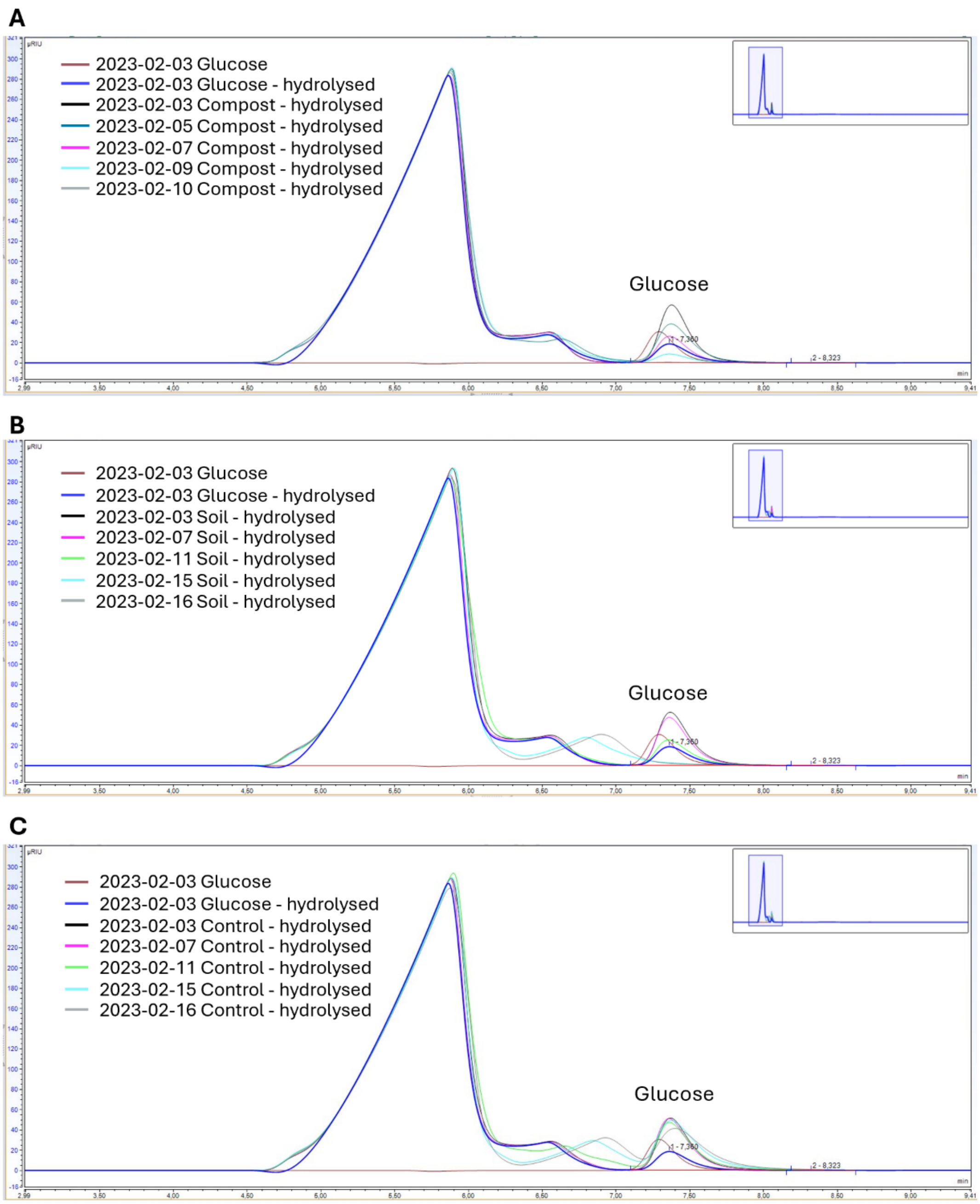


***Figure S2.2.*** Glucose depletion. Overlayed HPLC chromatograms of the analysed enrichment cultures to follow the glucose depletion over time. Whole broth samples were centrifuged, after which the remaining pullulan in the supernatant was hydrolysed by adding trifluoroacetic acid (TFA) to a final concentration of 4M. Samples were incubated at 100 °C for 4 hours. HPLC conditions are described in the method section. **A)** Compost enrichment (elevated temperature microbial source): inoculated with organic matter of a compost pile. **B)** Soil enrichment (temperature source control): inoculated with sediments from a cold water pond. **C)** Control: No inoculum.

## S3. Taxonomic composition of the enrichment cultures and the inoculum

A taxonomic classification of the compost inoculum (Figure S3) based on the metagenomics data has been performed. For illustration purposes, only the genera with ≥ 50 gene counts are depicted. The total amount of genera identified by taxonomic profiling equals 421, emphasizing the enormous variety of microorganisms present. A significant number of sequences remains unassigned. Therefore the taxonomic distribution might not be the best representation of the reality and needs to be further optimized. Nevertheless, it does show the enormous variety of microorganisms present in the inoculum.

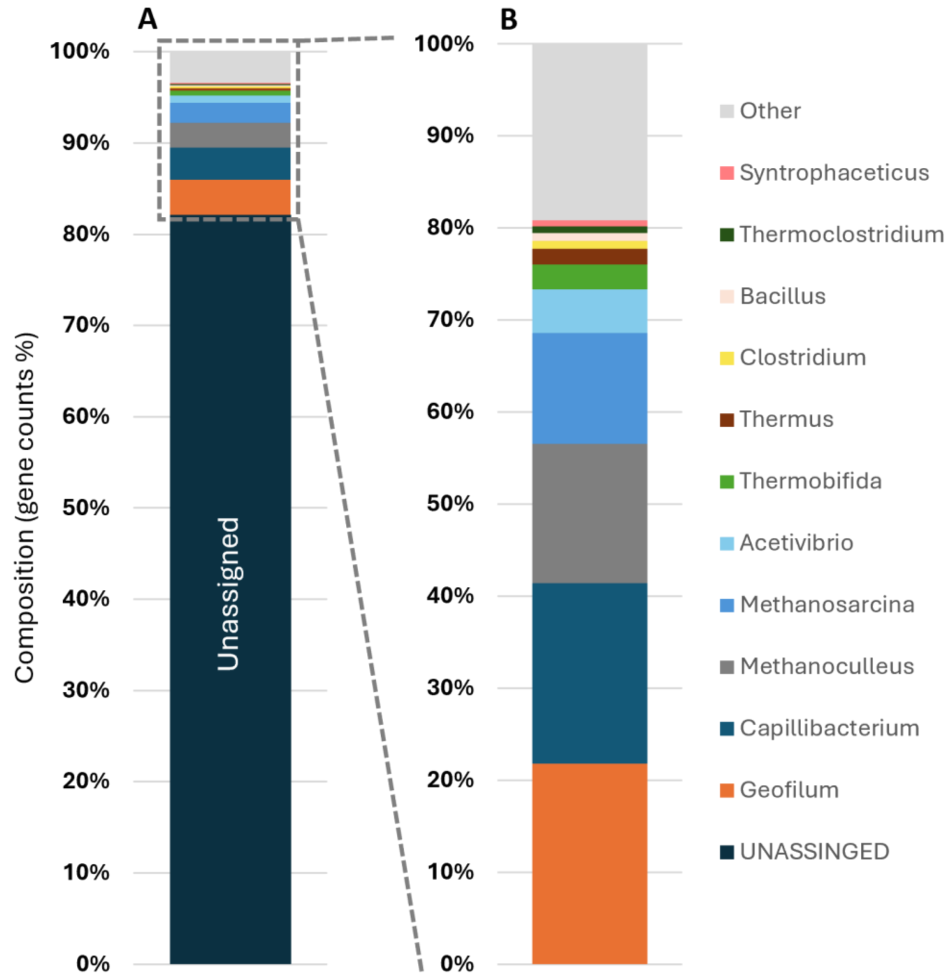


***Figure S3.1.*** Taxonomic composition of the compost inoculum based on gene count (metagenomics data). Taxonomic classification was performed on gene level. **A)** Including the unassigned sequences; **B)** Excluding the unassigned sequences.

In addition, a taxonomic classification of the enrichment cultures was performed based on the metaproteomics data (Figure S4).

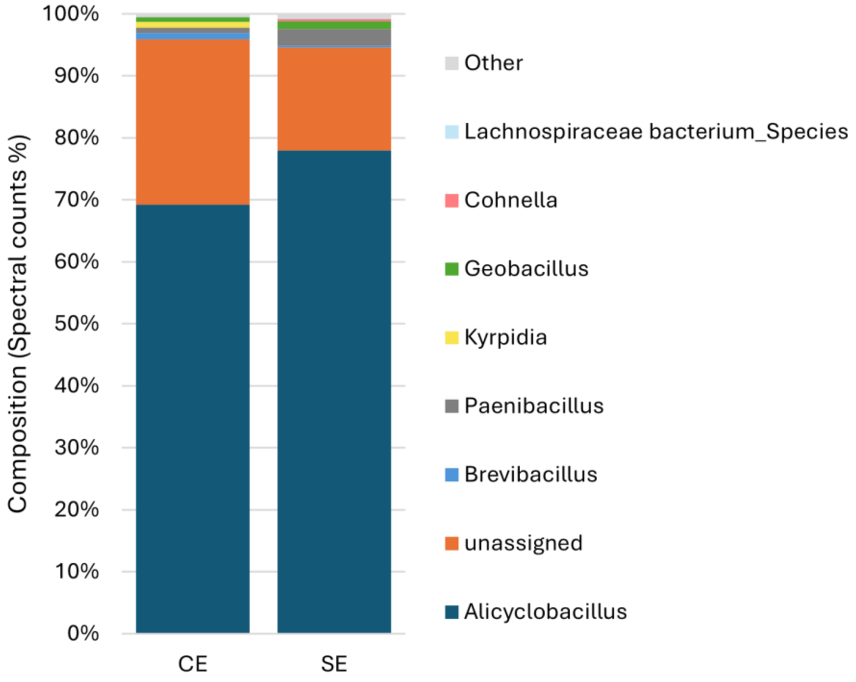


***Figure S3.2***. Taxonomic composition (genus level) of the 'Compost' and 'Soil' enrichments based on spectral counts (metaproteomics), normalized to 100%. **CE:** Compost enrichment; **SE:** soil enrichment.

## S4. Optimal temperature and pH of pullulan degrading enzymes

To provide an overview of the optimal temperature and pH of known pullulan degrading enzymes, the data obtained from the review by Kahar et al. (2022)[1] has been plotted in Figure S5.

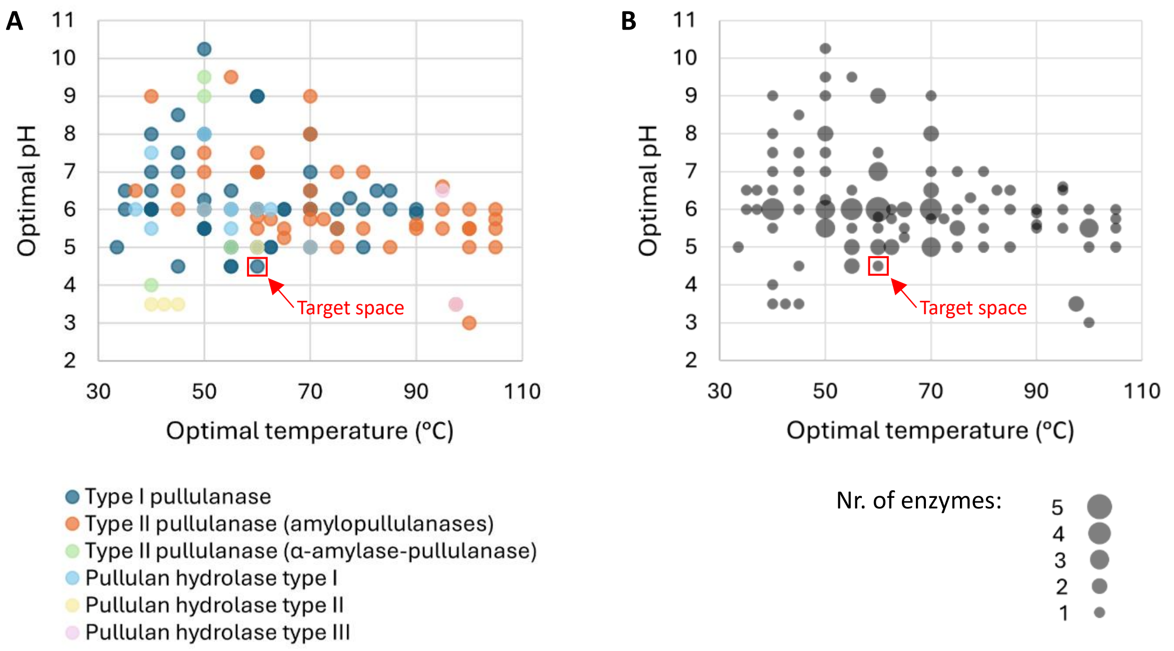


***Figure S4.*** Optimal temperature and pH of pullulan degrading enzymes. The data is obtained from the review by Kahar et al. (2022)[1]. **A)** Optimal temperature and pH of pullulan degrading enzymes categorized per enzyme group. Each dot represents a single enzyme. **B)** Optimal temperature and pH of pullulan degrading enzymes illustrated as a dot plot. The size of the dots represent the number of enzymes with a specified optimal temperature and pH.  The target space represent the enrichment culture conditions of this work (60 °C at pH 4.5).

## S5. Predicting cellular localization using SignalP and DeepLocPro

The final pullulan degrading enzyme candidates MMBJNONL_14124, MMBJNONL_07072 and MMBJNONL_12997 (Figure 4) were subjected to SignalP-6.0[5,6] and DeepLocPro[7] to predict the cellular localization of the enzymes and validate the cellular localization determined with proteomics (Table S1).

**Table S1.** Prediction of the cellular localization of the final pullulan degrading enzyme candidates using the bioinformatics tools SignalP-6.0[6] and DeepLocPro[7].

| Accession number | DeepLocPro localization (probability) | SignalP-6.0 (probability) |
|---|---|---|
| MMBJNONL_14124 | Extracellular (0.9240) | Yes (0.8539) |
| MMBJNONL_07072 | Extracellular (0.8952) | Yes (0.7402) |
| MMBJNONL_12997 | Cytoplasmic (0.9973) | No (0.9998) |

## S6. dbCAN3 annotation of the final pullulan degrading enzyme candidates

The final pullulan degrading enzyme candidates of the compost enrichment have been subjected to an annotation by dbCAN3[3] to validate the identification as CAZymes (Table S2, Figure S6). The detailed output of the analysis is provided in the SI Excel file 1.

**Table S2.** Overview dbCAN3 results. dbCAN3 annotation[3] has been performed on the final pullulan degrading enzyme candidates identified in the supernatant of the compost enrichment (see Table 1). EC 3.2.1.1: alpha-amylase; EC 3.2.1.41: Pullulanase; CMB34: Carbohydrate-Binding Module Family 34; GH13_39: Glycoside Hydrolase Family / Subfamily 39; GH53: Glycoside Hydrolase Family 53.

| Gene ID | EC# | HMMER | dbCAN_sub | DIAMOND | SignalP | # of Tools |
|---|---|---|---|---|---|---|
| MMBJNONL_14124 | 3.2.1.1\|3.2.1.41 | CBM34(395-538)+ GH13_39(621-970) | CBM34_e14+CBM34_e14+ GH13_e172 | CBM34+GH13_39 | Y(1-53) | 3 |
| MMBJNONL_07072 | 3.2.1.1\|3.2.1.41 | CBM34(394-538)+ GH13_39(621-842) | CBM34_e14+CBM34_e14+ GH13_e172 | CBM34+GH13_39 | Y(1-52) | 3 |
| MMBJNONL_05960 | - | GH53(187-524) | GH53_e21 | N | Y(1-33) | 2 |

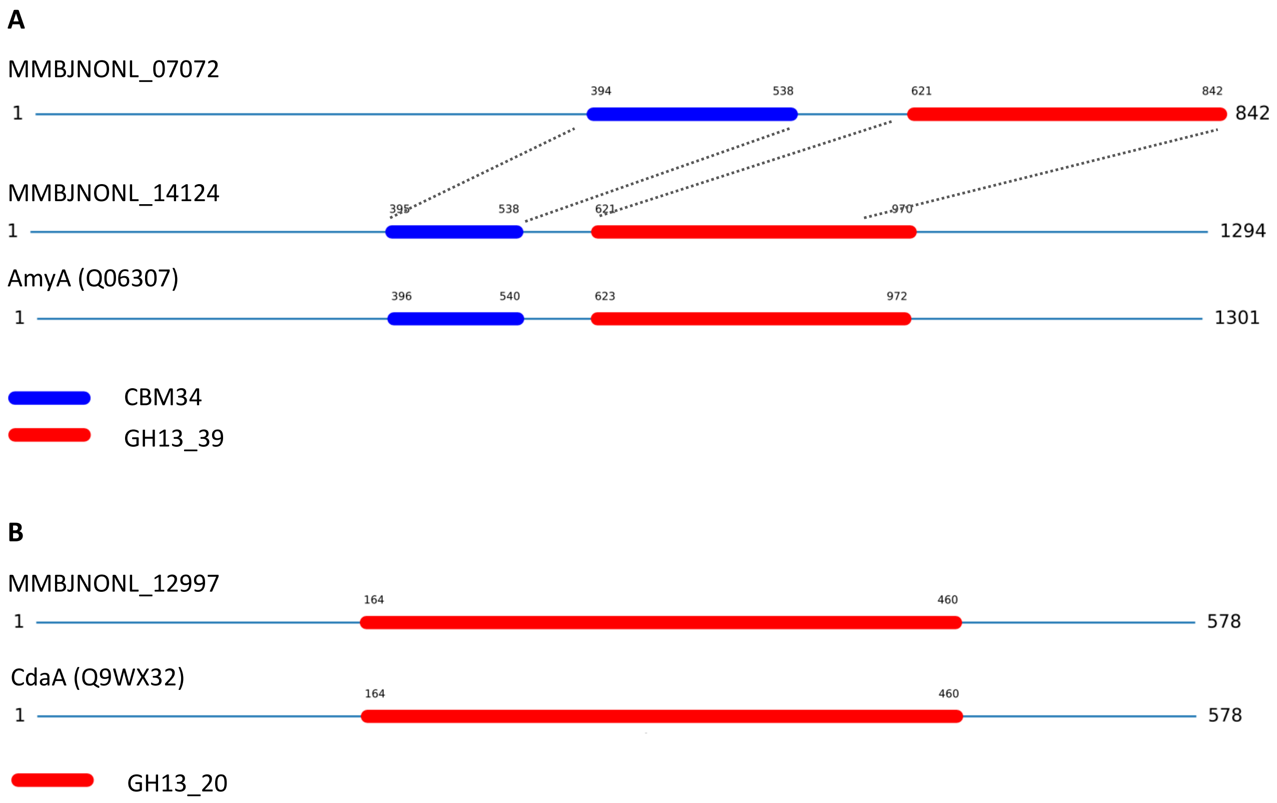


***Figure S6.*** Sequence annotation created with dbCAN3[3]. **A)** Sequence annotation of AmyA and the predicted extracellular pullulan degrading enzymes MMBJNONL_07072 and MMBJNONL_14124. **B)** Sequence annotation of CdaA and the predicted intracellular pullulan degrading enzyme MMBJNONL_12997.

## S7. Multiple sequence alignment

A multiple sequence alignment was performed both with a set of neopullulanases and the predicted intracellular pullulan degrading enzyme of this study and with a set of amylopullulanases and the predicted extracellular pullulan degrading enzymes of this study. The alignment was performed using the Constraint-based Multiple Alignment Tool (COBALT) of NCBI[2]. α-amylases are known to contain four conserved sequence regions, which are annotated in the alignments (Figure S7 and S8)[4,5].

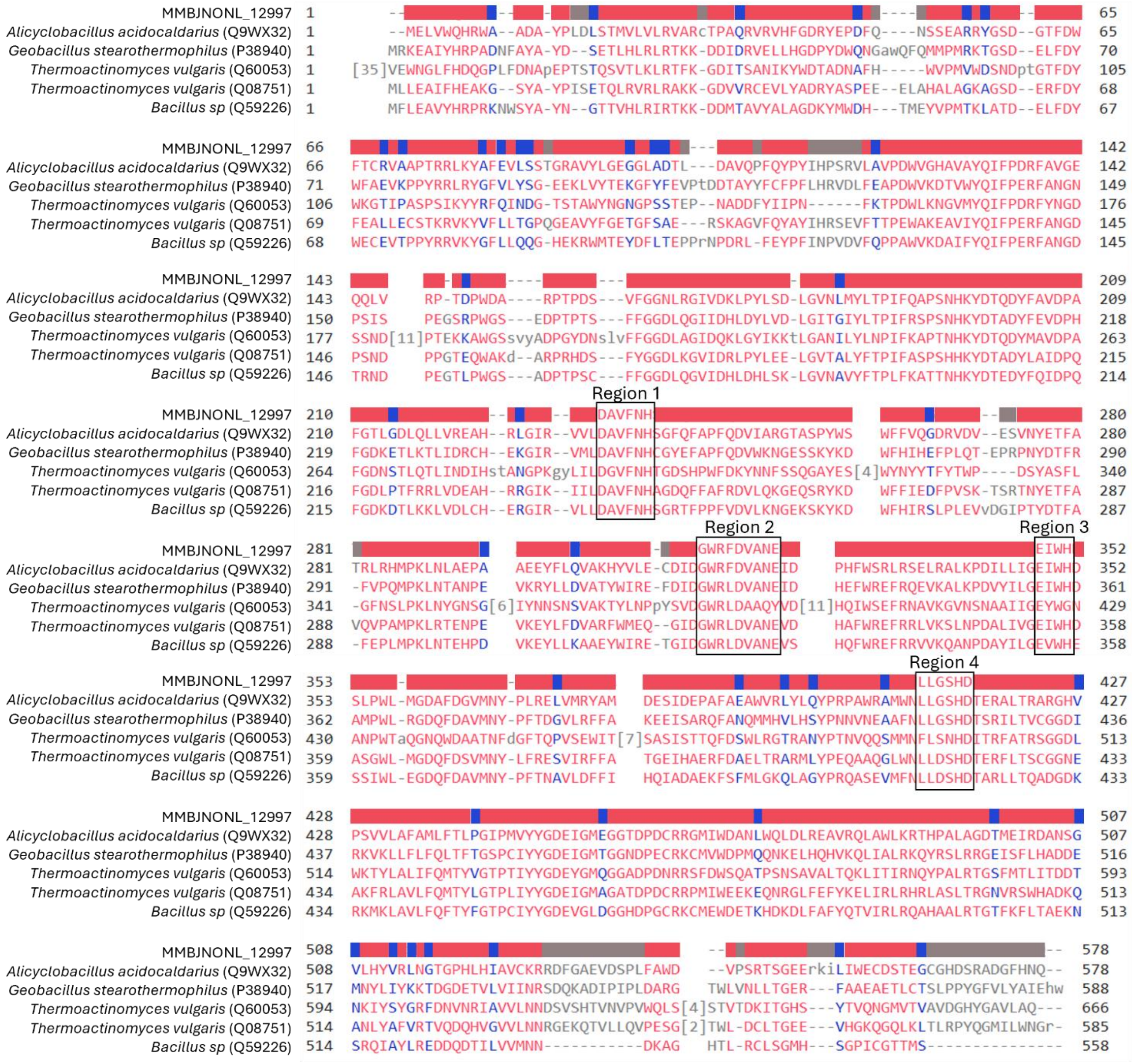


***Figure S7.*** Multiple sequence alignment of neopullulanases performed with the Constraint-based Multiple Alignment Tool (COBALT) from NCBI[2], using the default parameters. In red are the highly conserved amino acids, while in blue the lesser conserved amino acids. The known four conserved regions of alpha-amylases are outlined with a black box[4,5]. Uniprot identifiers of the used sequences are provided between brackets.

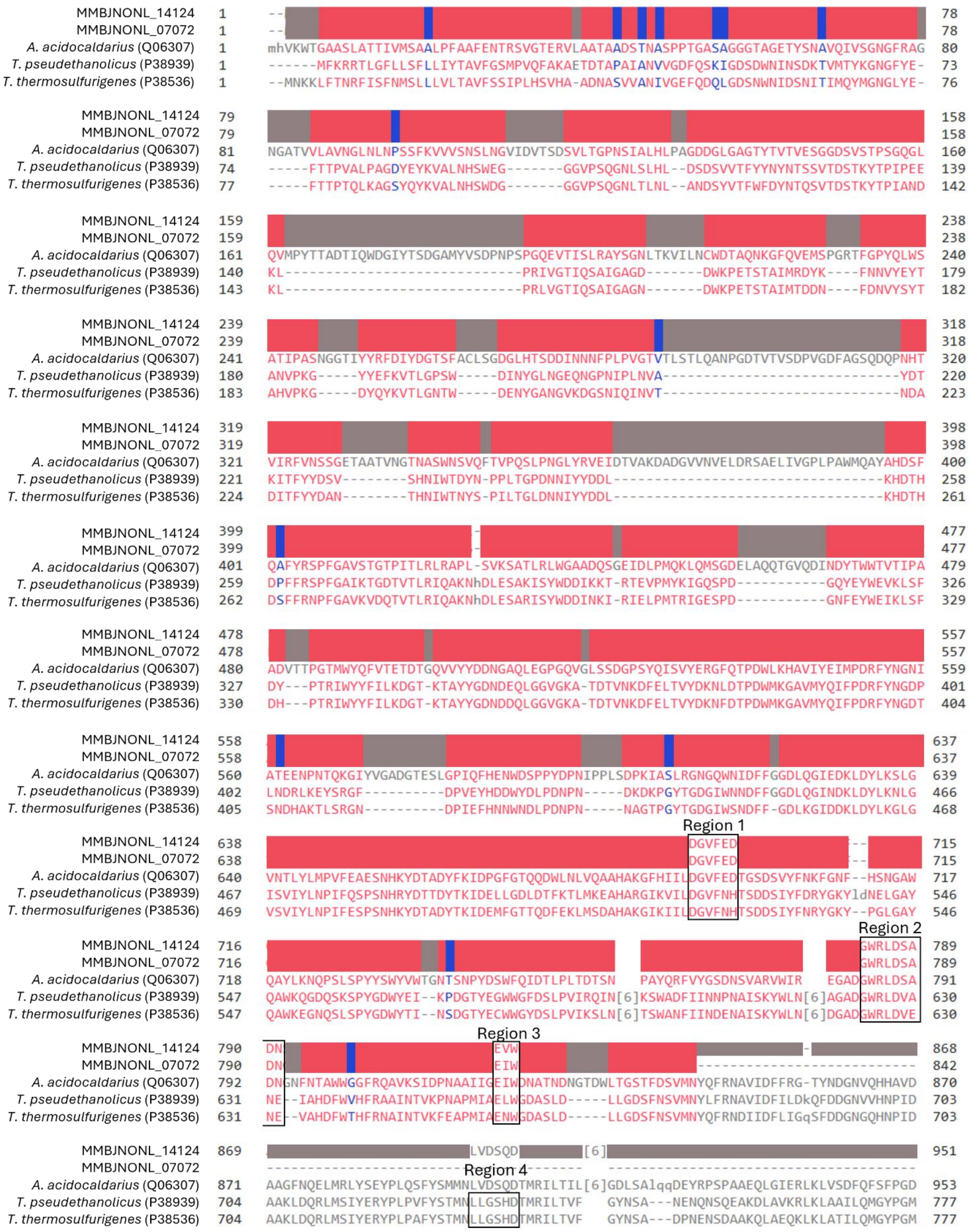


***Figure S8.*** Multiple sequence alignment of amylopullulanases performed with the Constraint-based Multiple Alignment Tool (COBALT) from NCBI[2], using the default parameters. In red are the highly conserved amino acids, while in blue the lesser conserved amino acids. The known four conserved regions of alpha-amylases are outlined with a black box[4,5]. Uniprot identifiers of the used sequences are provided between brackets. *A. acidocaldarius = Alicyclobacillus acidocaldarius; T. pseudethanolicus = Thermoanaerobacter pseudethanolicus; T. thermosulfurigenes = Thermoanaerobacterium thermosulfurigenes.*

Figure S8 Continued

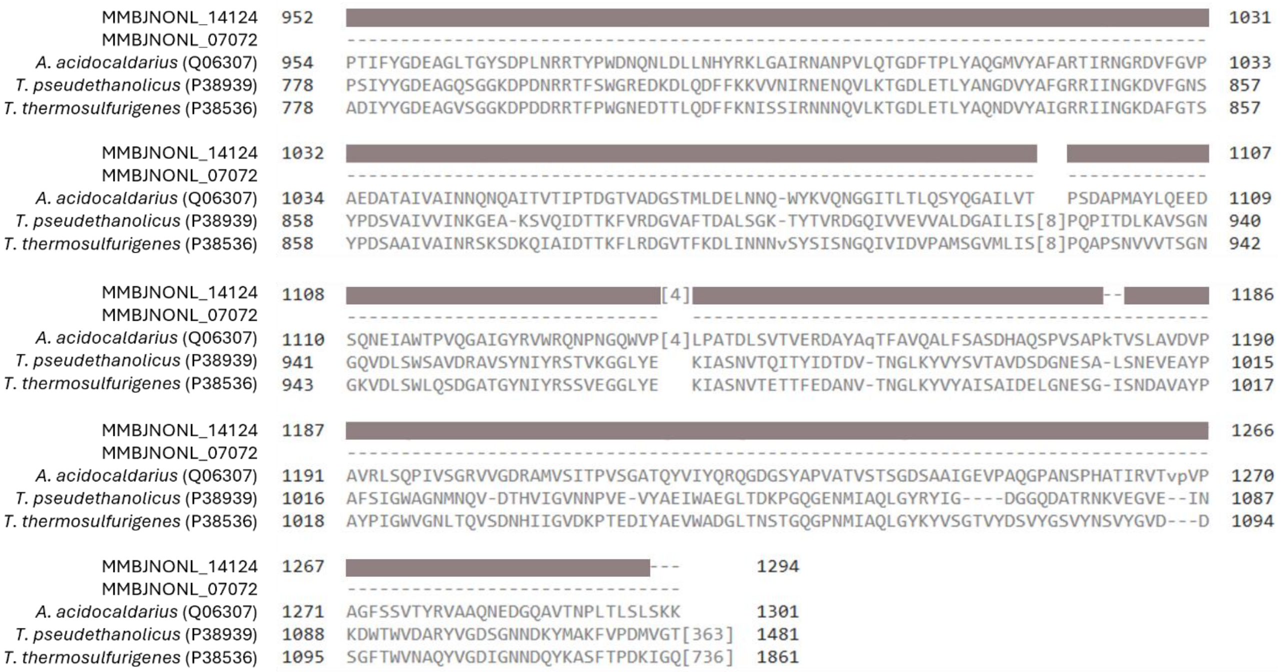

## S8. Expression and purification of MMBJNONL_14124 in *Rosetta2 DE3*

MMBJNONL_14124 was expressed with a C-terminal His-tag in *Rosetta2 DE3* by trenzyme GmbH (Germany). SDS-PAGE and western blot confirmed the successful soluble expression of the enzyme (Figure S9).

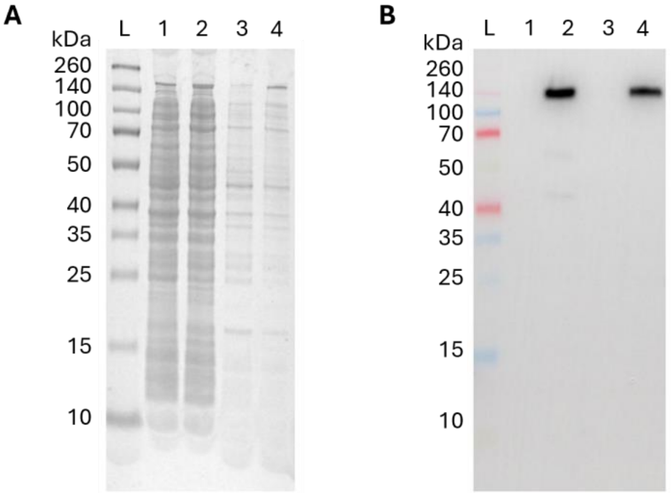


***Figure S9.*** **A)** 12% SDS-PAGE, Coomassie staining. **B)** 12% SDS-PAGE and anti-His-HRP (1:2000) Western blot (Standard detection; exposure time: 2 sec). The lanes for both gels were loaded **L)** protein ladder; **1)** pre induction, soluble; **2)** post induction, soluble; **3)** pre induction, insoluble; **4)** post induction, insoluble.

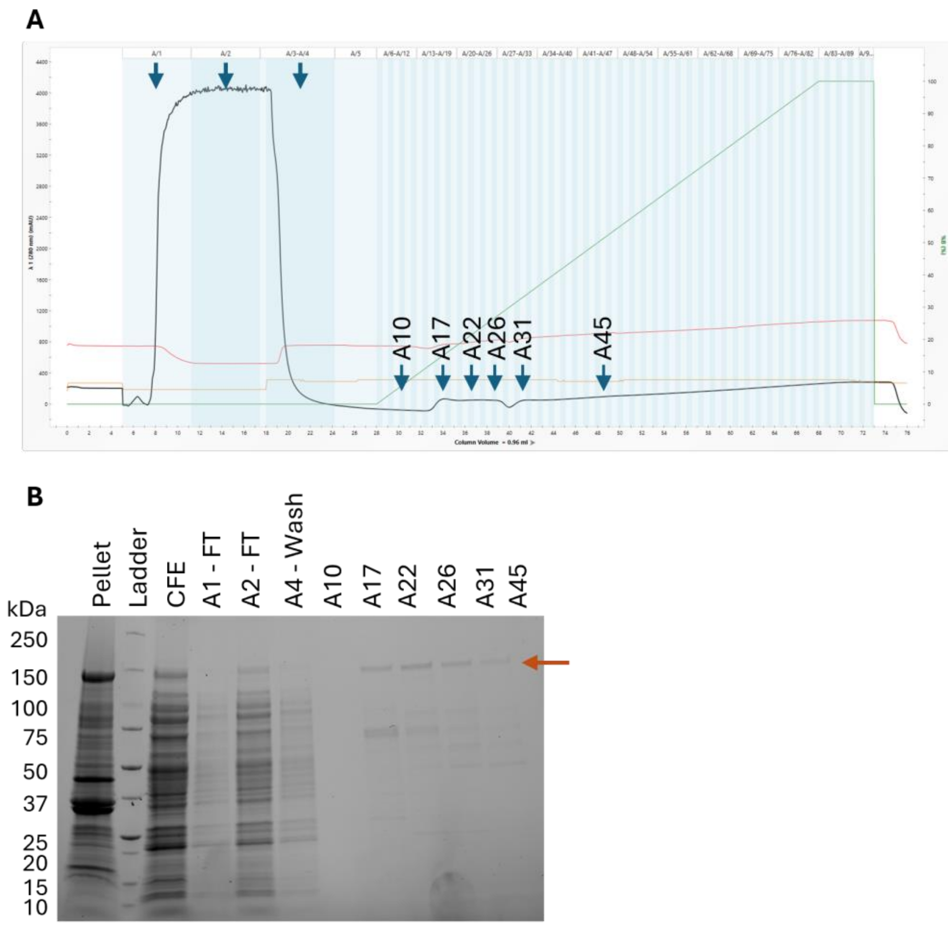


***Figure S10.*** **A)** UV chromatogram trace from MMBJNONL_14124 $Ni^{2+}$ affinity purification. **B)** SDS-PAGE (stain free) of $Ni^{2+}$ affinity purification fractions of MMBJNONL_14124. The lanes were loaded: **1)** Cell pellet after lysis; **2)** protein ladder; **3)** cell free extract; **4)** Flow through fraction A1; **5)** Flow through fraction A2; **6)** Wash fraction A4; **7)** Fraction A10; **8)** Fraction A17; **9)** Fraction A22; **10)** Fraction A26; **11)** Fraction A31; **12)** Fraction A45. The fraction correspond with the arrows in **A)**. The molecular weight of MMBJNONL_14124 equals 138.9 kDa and has been identified in the purified fractions A17, A22, A26 and A31. The orange arrow indicates the protein MMBJNONL_14124.

## S9. PGC-MS/MS based pullulan degrading activity assay

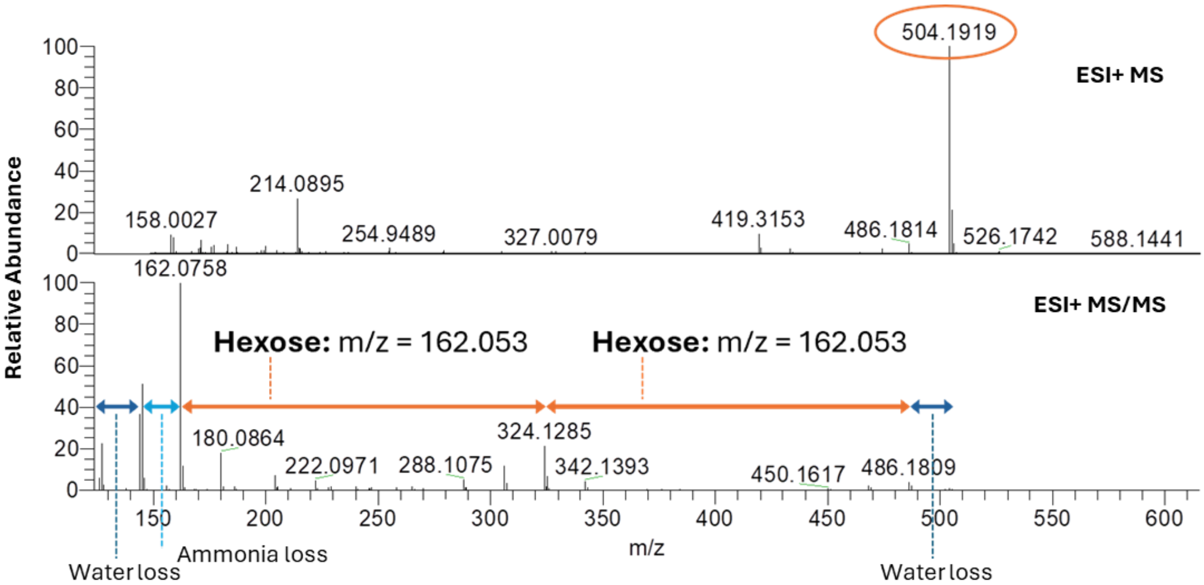


***Figure S11***. **Upper panel)** Summed mass spectrum over the retention time where the main hexotriose peak elutes after activity assay with the purified enzyme MMBJNONL_14124. **Lower panel)** Corresponding MS2 fragmentation spectrum of the observed hexotriose peak with the m/z of 504.1919. The fragmentation pattern reveals a successive loss of hexoses units (C6H10O5, 162.05282), terminated by an aminohexose (C6H12NO4+, 162.0761). The reducing end amino sugar was observed for all sugar residues, which converstion took place during the solid phase extraction and speed vac drying step which employed an ammonium bicarbonate buffer and elevated temperature over prolonged time periods.

# References


1 Kahar, U. M., Latif, N. A., Amran, S. I., Liew, K. J. & Goh, K. M. A bibliometric analysis and review of pullulan-degrading enzymes—past and current trends. *Catalysts* **12**, 143 (2022).

2 Papadopoulos, J. S. & Agarwala, R. COBALT: constraint-based alignment tool for multiple protein sequences. *Bioinformatics* **23**, 1073-1079 (2007). https://doi.org:10.1093/bioinformatics/btm076

3 Zheng, J. F. *et al.* dbCAN3: automated carbohydrate-active enzyme and substrate annotation. *Nucleic Acids Res* **51**, W115-W121 (2023). https://doi.org:10.1093/nar/gkad328

4 Lee, S. P., Morikawa, M., Takagi, M. & Imanaka, T. Cloning of the Aapt Gene and Characterization of Its Product, Alpha-Amylase-Pullulanase (Aapt), from Thermophilic and Alkaliphilic Bacillus Sp Strain Xal601. *Appl Environ Microb* **60**, 3764-3773 (1994). https://doi.org:Doi 10.1128/Aem.60.10.3764-3773.1994

5 Nakajima, R., Imanaka, T. & Aiba, S. Comparison of amino acid sequences of eleven different α-amylases. . *Applied Microbiology and Biotechnology* **23**, 355-360 (1986). https://doi.org:https://doi.org/10.1007/BF00257032

6 Teufel, F. *et al.* SignalP 6.0 predicts all five types of signal peptides using protein language models. *Nat Biotechnol* **40**, 1023-+ (2022). https://doi.org:10.1038/s41587-021-01156-3

7 Moreno, J., Nielsen, H., Winther, O. & Teufel, F. Predicting the subcellular location of prokaryotic proteins with DeepLocPro. *Bioinformatics* **40** (2024). https://doi.org:10.1093/bioinformatics/btae677
